# Discovery and resupply of pharmacologically active plant-derived natural products: A review

**DOI:** 10.1016/j.biotechadv.2015.08.001

**Published:** 2015-08-15

**Authors:** Atanas G. Atanasov, Birgit Waltenberger, Eva-Maria Pferschy-Wenzig, Thomas Linder, Christoph Wawrosch, Pavel Uhrin, Veronika Temml, Limei Wang, Stefan Schwaiger, Elke H. Heiss, Judith M. Rollinger, Daniela Schuster, Johannes M. Breuss, Valery Bochkov, Marko D. Mihovilovic, Brigitte Kopp, Rudolf Bauer, Verena M. Dirsch, Hermann Stuppner

**Affiliations:** a Department of Pharmacognosy, University of Vienna, Althanstrasse 14, 1090 Vienna, Austria; b Institute of Pharmacy/Pharmacognosy and Center for Molecular Biosciences Innsbruck (CMBI), University of Innsbruck, Innrain 80-82, 6020 Innsbruck, Austria; c Institute of Pharmaceutical Sciences, Department of Pharmacognosy, University of Graz, Universitätsplatz 4/I, 8010 Graz, Austria; d Institute of Applied Synthetic Chemistry, Vienna University of Technology, Getreidemarkt 9/163-OC, 1060 Vienna, Austria; e Institute of Vascular Biology and Thrombosis Research, Center of Physiology and Pharmacology, Medical University of Vienna, 1090 Vienna, Austria; f Institute of Pharmacy/Pharmaceutical Chemistry and Center for Molecular Biosciences Innsbruck (CMBI), University of Innsbruck, Innrain 80-82, 6020 Innsbruck, Austria; g Institute of Pharmaceutical Sciences, Department of Pharmaceutical Chemistry, University of Graz, Humboldtstrasse 46/III, 8010 Graz, Austria

**Keywords:** Natural products, Plants, Drug discovery, Phytochemistry, Pharmacology, Medicine, Ethnopharmacology, Computer modeling, Organic synthesis, Plant biotechnology

## Abstract

Medicinal plants have historically proven their value as a source of molecules with therapeutic potential, and nowadays still represent an important pool for the identification of novel drug leads. In the past decades, pharmaceutical industry focused mainly on libraries of synthetic compounds as drug discovery source. They are comparably easy to produce and resupply, and demonstrate good compatibility with established high throughput screening (HTS) platforms. However, at the same time there has been a declining trend in the number of new drugs reaching the market, raising renewed scientific interest in drug discovery from natural sources, despite of its known challenges. In this survey, a brief outline of historical development is provided together with a comprehensive overview of used approaches and recent developments relevant to plant-derived natural product drug discovery. Associated challenges and major strengths of natural product-based drug discovery are critically discussed. A snapshot of the advanced plant-derived natural products that are currently in actively recruiting clinical trials is also presented. Importantly, the transition of a natural compound from a “screening hit” through a “drug lead” to a “marketed drug” is associated with increasingly challenging demands for compound amount, which often cannot be met by re-isolation from the respective plant sources. In this regard, existing alternatives for resupply are also discussed, including different biotechnology approaches and total organic synthesis.

While the intrinsic complexity of natural product-based drug discovery necessitates highly integrated interdisciplinary approaches, the reviewed scientific developments, recent technological advances, and research trends clearly indicate that natural products will be among the most important sources of new drugs also in the future.

## 1. Introduction

For millennia, medicinal plants have been a valuable source of therapeutic agents, and still many of today's drugs are plant-derived natural products or their derivatives (Kinghorn et al., 2011; Newman and Cragg, 2012). However, since natural product-based drug discovery is associated with some intrinsic difficulties (discussed in more details in Section 2.1), pharmaceutical industry has shifted its main focus toward synthetic compound libraries and HTS for discovery of new drug leads (Beutler, 2009; David et al., 2015). The obtained results, however, did not meet the expectations as evident in a declining number of new drugs reaching the market (David et al., 2015; Kingston, 2011; Scannell et al., 2012). This circumstance revitalized the interest in natural product-based drug discovery, despite its high complexity, which in turn necessitates broad interdisciplinary research approaches (Heinrich, 2010a). In line with this, the Austrian “Drugs from Nature Targeting Inflammation (DNTI)” consortium was formed in 2008 uniting scientists with expertise in multiple disciplines relevant for natural product-based drug discovery. The DNTI program aimed at identifying and characterizing natural products with anti-inflammatory activity by the combined and synergistic use of computational techniques, ethnopharmacological knowledge, phytochemical analysis and isolation, organic synthesis, plant biotechnology, and a broad range of *in vitro*, cell-based, and *in vivo* bioactivity models [e.g., (Atanasov et al., 2013b; Fakhrudin et al., 2014; Schwaiberger et al., 2010); further details for the DNTI consortium are available at http://www.uibk.ac.at/pharmazie/pharmakognosie/dnti/]. Using their multidisciplinary expertise and the gathered experience from the DNTI participation, the authors of this review want to summarize here the currently established strategies and recent developments in the discovery and resupply of plant-derived bioactive natural products.

## 2. Plant-derived drug discovery: history, challenges, and significance

### 2.1. Natural products as drug candidates: a historical perspective

The first written records on medicinal applications of plants date back to 2600 BC and report the existence of a sophisticated medicinal system in Mesopotamia, comprising about 1000 plant-derived medicines. Egyptian medicine dates back to about 2900 BC, but its most useful preserved record is the “Ebers Papyrus” from about 1550 BC, containing more than 700 drugs, mainly of plant origin (Borchardt, 2002; Cragg and Newman, 2013; Sneader, 2005). Traditional Chinese medicine (TCM) has been extensively documented over thousands of years (Unschuld, 1986), and the documentation of the Indian Ayurveda system dates back to the 1st millennium BC (Patwardhan, 2005).

The knowledge on the medicinal application of plants in the Western world is mainly based on the Greek and Roman culture. Of particular importance are the compendia written by the Greek physician Dioscorides (1st century AD), and by the Romans Pliny the Elder (1st century AD) and Galen (2nd century AD) (Sneader, 2005). The Arabs preserved a large amount of the Greco-Roman knowledge during the Dark and Middle ages (i.e., 5th to 12th centuries), and complemented it with their own medicinal expertise, and with herbs from Chinese and Indian traditional medicines (Cragg and Newman, 2013). The invention of letterpress by Johannes Gutenberg led to a resurrection of the Greco-Roman knowledge in the 15th and 16th century, and to the compilation of several very influential herbal books that were widely distributed in Europe, like *The Mainz Herbal* (*Herbarius Moguntinus*, 1484) and *The German Herbal* (1485), both edited by Gutenberg's partner Peter Schöffer, the *Herbarium Vivae Eicones* (Otto Brunfels; 1530), the *Kreütter Buch* by Hieronymus Bock (1546) that was written in German, and *De Historia Stirpium* by Leonhart Fuchs that was published in Latin in 1542 and also in German in the following year (Sneader, 2005).

During all that time, medicinal plants were only applied on an empirical basis, without mechanistic knowledge on their pharmacological activities or active constituents. It was only in the 18th century that Anton von Störck, who investigated poisonous herbs such as aconite and colchicum, and William Withering, who studied foxglove for the treatment of edema, laid the basis for the rational clinical investigation of medicinal herbs (Sneader, 2005).

Rational drug discovery from plants started at the beginning of the 19th century, when the German apothecary assistant Friedrich Sertürner succeeded in isolating the analgesic and sleep-inducing agent from opium which he named *morphium* (morphine) after the Greek god of dreams, Morpheus. He published a comprehensive paper on its isolation, crystallization, crystal structure, and pharmacological properties, which he studied first in stray dogs and then in self-experiments (Sertürner, 1817). This triggered the examination of other medicinal herbs, and during the following decades of the 19th century, many bioactive natural products, primarily alkaloids (e.g., quinine, caffeine, nicotine, codeine, atropine, colchicine, cocaine, capsaicin) could be isolated from their natural sources (Corson and Crews, 2007; Felter and Lloyd, 1898; Hosztafi, 1997; Kaiser, 2008; Kruse, 2007; Sneader, 2005; Zenk and Juenger, 2007). Apothecaries who specialized in the purification of these compounds were the progenitors of pharmaceutical companies. The first one was H.E. Merck in Darmstadt (Germany) who started extracting morphine and other alkaloids in 1826 (Kaiser, 2008). Subsequently, efforts were undertaken to produce natural products by chemical synthesis in order to facilitate production at higher quality and lower costs. Salicylic acid was the first natural compound produced by chemical synthesis in 1853 (Kaiser, 2008).

After the discovery of penicillin (1928), an era of drug discovery from microbial sources was initiated in the 1930s, that laid the scientific and financial foundation of the modern pharmaceutical industry after World War II. At that time, the therapeutic use of extracts and partly purified natural products was increasingly replaced by the use of pure compounds (Beutler, 2009; David et al., 2015). Despite the advent of combinatorial chemistry and HTS campaigns during the last decades, the impact of natural products for drug discovery is still very high. Of the 1073 new chemical entities belonging to the group of small molecules that had been approved between 1981 and 2010, only 36% were purely synthetic, while more than the half were derived or inspired from nature (Newman and Cragg, 2012). A substantial number of these compounds have been discovered in higher plants (Kinghorn et al., 2011). Particularly prominent examples of plant-derived natural compounds that have become indispensable for modern pharmacotherapy can be found in the field of anti-cancer agents, e.g., paclitaxel and its derivatives from yew (*Taxus*) species, vincristine and vinblastine from Madagascar periwinkle (*Catharanthus roseus* (L.) G. Don), and camptothecin and its analogs initially discovered in the Chinese tree *Camptotheca acuminata* Decne. (Cragg and Newman, 2013; Kinghorn et al., 2011). Further notable examples include the cholinesterase inhibitor galanthamine that has been approved for the treatment of Alzheimer's disease and was initially discovered in *Galanthus nivalis* L. (Mashkovsky and Kruglikova-Lvova, 1951), and the important antimalarial and potential anti-cancer agent artemisinin originally derived from the traditional Chinese herb *Artemisia annua* L. (Klayman et al., 1984).

### 2.2. Challenges contributing to the decline of plant-derived natural products as drug discovery source

Since very often plants are collected directly from their natural habitat, the correct identification and nomenclature are essential and the basis for all following steps. For an unambiguous identification, a combination of methods might be necessary, such as genetic and chemical analysis in addition to morphological and anatomical characterization (Bucar et al., 2013). Continuously ongoing modifications in plant taxonomy as well as synonymy issues add to the difficulty of this challenging task (David et al., 2015). Moreover, collection of the plant material and accurate documentation, botanical identification, as well as preparation of the herbarium vouchers are tasks that cannot be automated (David et al., 2015) and need specialists who become increasingly rare (Bucar et al., 2013).

Important challenges related with the use of plants as a source for identification of bioactive compounds are related with the accessibility of the starting material. Often the available amount of natural products is low. Although many plant-derived natural products have already been isolated and characterized, available compound quantities are often insufficient for testing for a wide range of biological activities. While small amounts of plant material are usually required for an initial pharmacological evaluation, much larger quantities are needed for through characterization of the pharmacological activity of its constituents. Furthermore, limited availability becomes even more problematic when a bioactive plant-derived natural product is identified to have a very promising bioactivity and becomes a pharmaceutical lead. Recollections of wild species may turn difficult, since plant habitats can rapidly disappear under anthropic pressure. Moreover, the habitat of plants, particularly of protected species, needs to be respected when collecting from the wild (David et al., 2015), and season-dependent chemical composition of plant material may limit the time window for recollection (e.g., blossom collection necessitates collection during the flowering season). In cases of imported plant material, also an entire array of additional factors might affect its accessibility, for example local wars, natural catastrophes, or changing legal regulations for cross-border traveling and export of plant material. The importance of plant material accessibility is illustrated by a recent study (Amirkia and Heinrich, 2014) investigating the correlation between species abundance of alkaloids occurrence and their use as pharmaceutical drugs. Species distribution was assessed on the basis of Global Biodiversity Information Facility (GBIF) data. The authors found that 93% of all alkaloids in medical use have more than 50 occurrences in the GBIF database, and only two have less than 10 occurrences. Therefore, the authors conclude that natural products occurring in many different species are more favorable for medicinal use, and that supply constraints are a major obstacle to the successful research, development and commercialization of natural products.

In many cases, when a plant becomes commercialized as a herbal medicine or when one of its constituents starts getting used as a pharmaceutical drug, its populations become threatened due to extensive wildcrafting and unsustainable harvesting techniques (Cordell, 2011; Vines, 2004). The classical example for this compound supply problem was the so-called “taxol supply crisis” (Cragg et al., 1993; Kingston, 2011). When the compound turned out to possess remarkable clinical efficacy in ovarian cancer, suddenly the demand for taxol increased tremendously. However, at that time, the compound was only accessible from the bark of the western yew (*Taxus brevifolia* L.). This was on one hand problematic because the whole production process including tedious bark collection and drying, extraction, and purification was very time-consuming. On the other hand, concerns on the ecological impact of intensive bark collection were raised (Cragg et al., 1993; Kingston, 2011). Although taxol is meanwhile accessible via alternative routes (see Section 4.2), the problem of sustainable supply of herbal material still frequently occurs. Cultivation would be a more sustainable alternative to wildcrafting, nevertheless, approximately two thirds of the 50,000 medicinal plant species used world-wide are still wildcrafted (Canter et al., 2005). Therefore, institutions like WHO (WHO, 2003) and European Medicines Agency (EMA) (EMA, 2006) developed guidelines on good agricultural and collection practices (GACP) for medicinal plants in order to promote sustainable plant collection techniques and to reduce the ecological problems produced by wildcrafting of medicinal plants.

Apart from that, ecological and legal considerations also have an important influence on accessibility of plants as a source of drug discovery, especially laws dealing with plant access and sharing of benefits, as well as patentability issues with local governments in the countries of origin. The United Nation's Convention on Biological Diversity (CBD; http://www.cbd.int/doc/legal/cbd-en.pdf), signed in 1992 by the international community in Rio de Janeiro, Brazil, aims at: (1) conserving the biodiversity; (2) sustainably using its genetic resources; and (3) sharing the benefits from their use in a fair and equitable manner (Cragg et al., 2012; Kingston, 2011; Soejarto et al., 2004). Resulting from CBD, the provider countries, their people and representatives also become important stakeholders that need to be considered in plant-based drug discovery programs (Heinrich, 2010a). Although the CBD provided a framework for countries to regulate and define bioprospecting, the treaty left many open questions, particularly in the issue of access and benefit sharing (Cragg et al., 2012; Kingston, 2011). On one hand, CBD could not always rebut the skepticism toward bioprospecting in many developing countries due to previous exploitation of their biodiversity; some countries issued very stringent protective regulations, and some were very slow in establishing the necessary legal framework, leading to confusion where to go for permissions and who had authority to grant them. On the other hand, the expectations of biodiversity-rich countries on the potential monetary benefits to achieve from drugs developed from their genetic resources were highly exaggerated, if one considers that from the 114,000 extracts derived from 12,000 species that the US National Cancer Institute (NCI) investigated over decades, only taxol and camptothecin are currently used as pharmaceutical drugs. These issues frequently hampered the access to samples from biodiversity-rich countries in the last two decades and thereby discouraged pharmaceutical companies from natural product-based drug discovery (Cragg et al., 2012; David et al., 2015; Kingston, 2011). In order to improve this unfavorable situation, the Nagoya Protocol on access to genetic resources and the fair and equitable sharing of benefits arising from their utilization to the convention on biological diversity (http://www.cbd.int/abs/doc/protocol/nagoya-protocol-en.pdf), has been published in 2011 and has come into force in October 2014, after reaching ratification by 50 countries. The protocol is legally binding and particularly aims at bringing more clarity into questions of access and benefit sharing (Burton and Evans-Illidge, 2014; Oliva, 2011). Although some researchers are worried that the protocol might lead to stricter regulations that could hamper drug discovery and even be counterproductive for biodiversity conservation (Gilbert, 2010), others expect that – provided that the protocol is implemented into national laws in a sensitive way – the higher legal certainty will revitalize the interest to investigate plants from biodiversity-rich countries, and thereby provide incentives to conserve biodiversity (Burton and Evans-Illidge, 2014; Cragg et al., 2012).

Besides the accessibility of the plant material, also its quality is of great importance. Available plant material often varies on quality and composition and this can hamper the assessment of its therapeutic claims. The chemical composition is not only dependent on species identity and harvest time, but also on soil composition, altitude, actual climate, processing, and storage conditions. Moreover, during extraction, as well as during the isolation processes, transformation and degradation of compounds can occur (Bucar et al., 2013; Jones and Kinghorn, 2012). Another aspect determining the chemical composition of the starting plant material is that endophytic organisms, such as fungi and bacteria, might inhabit plants. As a result, natural products present in the collected plant material might be in some occasions metabolites of the endophytic organism, or plant products induced as a result of the interaction with this organism (David et al., 2015).

Further complications related to the resupply of bioactive natural products arise from the fact that natural products are more likely to have complex chemical structures with numerous oxygen-containing substituents and chiral centers, which hampers the development of methods for total synthesis or derivatization that might be needed for property optimization of drug candidates. In contrast, pharmaceutical leads originating from synthetic libraries are usually comparably easy to generate and modify using simple chemical approaches (Butler, 2004; Henrich and Beutler, 2013; Li and Vederas, 2009).

Another major challenge for natural product drug discovery programs is common incompatibility of natural products with HTS (Koehn and Carter, 2005). Investigation of a large number of plant extracts by HTS, followed by the identification and characterization of bioactive constituents is highly challenging. Adaption and changes of sample preparation and assay designs are necessary in order to apply HTS for bioactivity detection of plant extracts and to identify potent pure compounds thereof. In general, HTS can be conducted using cell-free or cell-based assays. It requires high reproducibility, accuracy, robustness, and reliable liquid handling systems. Test compounds should not decompose or precipitate, should not interfere with assay reagents nor show non-specific effects. Especially natural products often fail in fulfilling these requirements. The maintenance of plant extracts integrity might be very problematic due to their complexity. Extracts often show high viscosity, tend to aggregate or precipitate, or contain components that non-specifically bind proteins, which can result in misleading assay outcomes, therefore necessitating sophisticated sample preparation or fractionation of the crude extracts prior to testing (Coan et al., 2011; Johnson et al., 2011; Maes et al., 2012; Schmid et al., 1999; Tu et al., 2010). Natural product extracts are also likely to contain fluorescent or fluorescence quenching compounds, which interfere with the fluorescent HTS endpoint measurements, whereby the presence of colored compounds might also interfere with colorimetric HTS endpoints (Gul and Gribbon, 2010; Henrich and Beutler, 2013; Zou et al., 2002). Moreover, plant extracts may contain compound classes that are obstructive for certain assay types and might lead to false positive or false negative results. Particularly common highly apolar compounds, such as fatty acids (Balunas et al., 2006), common polar compounds, such as polyphenols and flavonoids (Zhu et al., 1997, 2013; Zou et al., 2002), as well as chlorophyll (Henrich et al., 2006) might be especially problematic since they can interfere with a range of different assays. Much effort is necessary to remove such constituents from samples prior to testing (Cardellina et al., 1993; Collins et al., 1998; Picker et al., 2014) or to modify the assay system in order to avoid their detection (Sasiela et al., 2008). Next to organic molecules, also some inorganic constituents, such as metals, can lead to false positive results in HTS (Hermann et al., 2013). This might be especially problematic for HTS of plant extracts, since many plants concentrate metals from their environment (Fernando et al., 2013), and metal impurities could be present in commercially available plant samples (Eisenberg et al., 2011). Furthermore, cytotoxic constituents might cause problems in cell-based assays, since they can mask the detection of other bioactivities or the presence of other compounds with the desired efficacy. Saponins, for example, which possess detergent effects, can lead to the lysis of cells and therefore interfere with the results of cell-based assays (Henrich and Beutler, 2013). For further details on the application of HTS to natural product samples and its challenges, the reader is referred to the excellent and comprehensive recent review by Henrich and Beutler (Henrich and Beutler, 2013).

Further difficulty is set by the fact that determination of the precise molecular mechanism of action of natural products is a challenging task [e.g., curcumin, triptolide; (Corson and Crews, 2007)]. However, a detailed knowledge of the interaction of a drug candidate compound with its molecular target is very advantageous for the drug development process, because it allows property optimization by medicinal chemistry approaches, and on some occasions a more appropriate clinical trial design.

The conduction of rigorous clinical trials needed for approval of natural products as drugs represents another major difficulty. While such clinical trials are often feasible just with industrial support due to the high costs, at the same time the interest of pharmaceutical companies in natural products that are not synthetically modified is limited due to controversies with their patentability [e.g., curcumin; (Corson and Crews, 2007)]. In this line, the recent situation concerning patentability of natural products got even more difficult after new guidelines were issued on 4th of March 2014 by the United States Patent and Trademark Office (“*Guidance for Determining Subject Matter Eligibility of Claims Reciting or Involving Laws of Nature, Natural Phenomena, & Natural Products*”). The new guidelines state that a patent claim must demonstrate a “marked difference” from a known natural law, material, or phenomenon, and their issuing came after two relevant high-profile Supreme Court decisions: *The Association for Molecular Pathology versus Myriad*, which ruled that isolated and purified DNA is not patentable, and *Mayo versus Prometheus*, which ruled that methods for determination of optimal drug doses based on levels of a naturally occurring metabolite is not eligible for patenting (Harrison, 2014). Aside of issues related to patentability of natural products, it should be also noted that there has been some general shift in the pharmaceutical industry from small molecule-based drug discovery toward biological big molecules (biologicals; e.g., therapeutic proteins or nucleic acids) (Appendino et al., 2010). However, the patient costs for biologicals are much higher than the costs for small molecule drugs, and their increasing use is putting a rising pressure on national health insurances. Furthermore, the high prices of this type of pharmaceuticals is not expected to quickly drop in the near future after patents expiration, because biological generics (biosimilars) require additional clinical bioequivalence studies to be approved for use and overall have significantly higher development and manufacturing costs in comparison to small molecule generics. Therefore, to relive the increasing financial burden, turning back to some “old” small molecule-based approaches is conceivable (Appendino et al., 2010).

Due to the challenges described above, the interest in natural product-based drug discovery has been gradually declining. Even in the last decade, many big and medium-sized pharmaceutical companies, which were still active in the area in the 1990s, terminated their natural product programs, leaving natural product research to a major extent to academic universities and start-up companies (Beutler, 2009; David et al., 2015; Ortholand and Ganesan, 2004).

### 2.3. Renewal of the interest in natural product-based drug discovery

The results obtained by HTS of large synthetic compound libraries, which were introduced in the 1990s (Scannell et al., 2012), did not meet the expectations. Instead of introducing more drugs to the market, the approval rates of new drugs declined. Whereas 45 new drugs were approved by the US Food and Drug Administration (FDA) in 1990, only 21 were approved in 2010 (David et al., 2015; Kingston, 2011). While the reasons for this declining trend are complex (Kola and Landis, 2004), one important aspect is that synthetic compound libraries usually cover only a small range of the chemical diversity. Moreover, due to similar generation strategies, the HTS-compound libraries of different companies often overlap. Due to high sample numbers in such libraries, compounds to be investigated further are often selected quickly, mainly based on potency values (Scannell et al., 2012), although a negative correlation of cell-free *in vitro* potency and favorable ADME/T (absorption, distribution, metabolism, excretion/toxicity) is likely (Gleeson et al., 2011).

The plant kingdom includes a high number of species, producing a diversity of bioactive compounds with different chemical scaffolds. According to previous estimations only 6% of existing plant species have been systematically investigated pharmacologically, and only around 15% phytochemically (Cragg and Newman, 2013; Fabricant and Farnsworth, 2001; Verpoorte, 1998, 2000). Although today the percentage of better characterized species is likely higher due to ongoing research efforts, it is still conceivable that there is a huge number of plant compounds that are not well investigated pharmacologically in the approximately 310,000 plant species described so far (IUCN, 2015). Unfortunately, as a result of ongoing climate changes and anthropogenic factors, a significant decrease in global vegetative species in the next years is predicted (Maclean and Wilson, 2011; Thomas et al., 2004), endangering the sources of potential new drugs from nature and prompting urgent actions.

Since natural products are made from living organisms, they possess properties that are evolutionary optimized for serving different biological functions (e.g., binding to specific target proteins or other biomolecules) (Appendino et al., 2010; Hunter, 2008). Detailed analyses of structural differences between natural products and molecules generated by combinatorial synthesis found that major differences originate from the introduction of properties making combinatorial synthesis more efficient. For example, chiral separation is challenging and expensive. Therefore, creating molecules with a low number of chiral centers is favorable. Besides a much lower number of chiral centers, synthetic compounds tend to have a lower molecular weight, a higher number of freely rotatable bonds, higher chain lengths, a lower number of rings, less oxygen but more nitrogen, sulfur, and halogen atoms, a lower number of Lipinski-type H-bond acceptors and donors, and higher calculated octanol-water partition coefficients (cLogP values). Other differences are the complexity of ring systems and the degree of saturation (Feher and Schmidt, 2003; Koehn and Carter, 2005; Lee and Schneider, 2001; Stahura et al., 2000). These structural differences, especially the significantly lower number of chiral centers, the lower size, and the higher flexibility result in weaker and less specific activity of synthetic compounds (Feher and Schmidt, 2003; Klebe, 2009). On the contrary, natural products often have selective biological actions due to binding affinities for specific proteins relevant for their biological functions, possess superior chemical diversity and complexity developed during biosynthesis (Clardy and Walsh, 2004; Koehn and Carter, 2005), and often have more advantageous ADME/T properties.

Particularly in the context of drug discovery from medicinal plants, a big advantage is that sometimes well documented ethnopharmacological information about the traditional use is available, which can provide hints for compounds therapeutically effective in humans (Corson and Crews, 2007; Heinrich, 2010a; Heinrich and Gibbons, 2001; Kinghorn et al., 2011). In line with this notion, analysis of 122 plant-derived compounds identified to be globally used as drugs revealed that 80% of them originate from plants that have ethnomedical use identical or related to the indications for which the respective pure compounds are prescribed (Fabricant and Farnsworth, 2001; Farnsworth et al., 1985). Furthermore, it was even demonstrated that natural products used for the development of medicines are highly likely to be used traditionally, even if this was not known at the stage of drug development (e.g., the discovery of the anti-cancer agent taxol from *T. brevifolia* was done with a random screening approach, but later on it came to light that the plant has been used by western Indian cultures as a medicine) (Heinrich, 2010a; Moerman, 1998). Importantly, knowledge about traditionally used medicines is disappearing even faster than the biodiversity of plant species and with the current tempo of globalization, much valuable information is on the risk of getting lost forever (Appendino et al., 2010).

Resulting from the above discussed advantages of natural products, in spite of the predominant industrial focus on HTS approaches with synthetic compound libraries, natural products still represent a valuable source for drug discovery (Newman and Cragg, 2012). Plant-derived natural products approved for therapeutic use in the last thirty years (1984–2014) are summarized in Table 1. As evident from the table, these plant-derived natural products are modulating a diverse range of molecular targets and are used for the treatment of various disease conditions. As a snapshot of the current state and perspective for future developments, overview of advanced plant-derived small chemical entities that are in actively recruiting clinical trials are presented in Table 2.

**Fig. 1:**
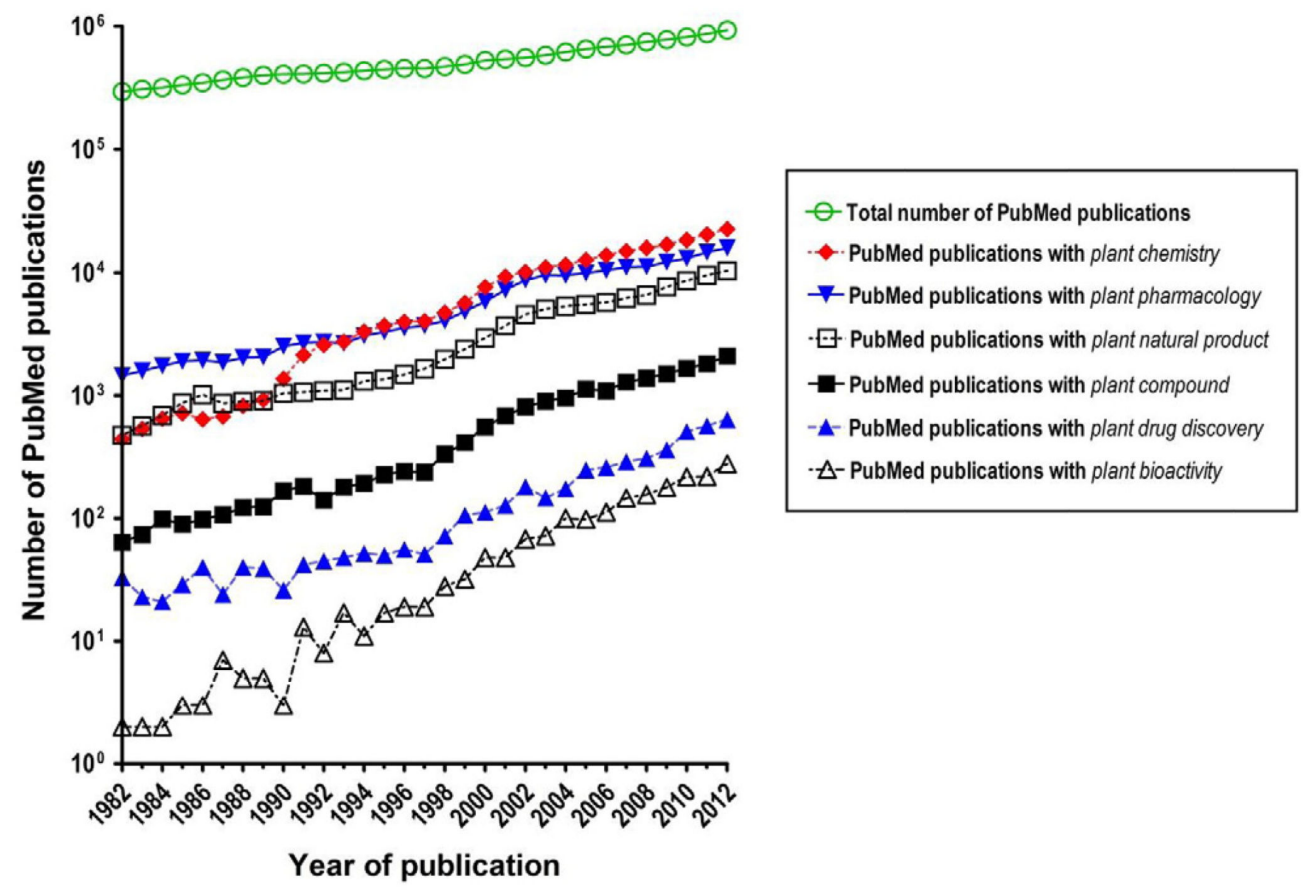
PubMed publication trend analysis, demonstrating increased scientific interest in plant-derived natural product pharmacology, chemistry, and drug discovery. The data were retrieved with MEDSUM (http://webtools.mf.uni-lj.si/public/medsum.html) on 15th of June 2015, and cover the time period 1982–2012 (newer data are not included because of the lack of coverage). As indicated, the used search keywords were *plant chemistry, plant pharmacology, plant natural product, plant compound, plant drug discovery, plant bioactivity*, and the total number of PubMed publications per year was retrieved by search with the symbol *. The trend analysis reveals that the increase of PubMed citations in the target areas is faster than the increase in the total number of annual PubMed citations (indicated by the steeper slopes of the respective trend lines).

Reflecting better appreciation of the advantages of natural products and the growing interest in plant-derived natural product-based drug discovery, a rapid increase in the number of scientific studies targeting this research area is observed upon analysis of the recent PubMed publication trends (Fig. 1; data retrieved with MEDSUM, http://webtools.mf.uni-lj.si/public/medsum.html). The revived scientific interest in plant-derived natural product-based drug discovery is paralleled with major scientific and technological advances in the relevant research fields, including better understanding of diseases and their underlying mechanisms, advances in screening methods and analytical equipment, increasing number of targets available for testing, and improved possibilities for optimization of natural leads using synthetic modification strategies [reviewed in (Henrich and Beutler, 2013; Koehn and Carter, 2005; Li and Vederas, 2009; Paterson and Anderson, 2005)].

## 3. Approaches for the discovery of new pharmacologically active plant compounds

### 3.1. Approaches to selecting starting material

In the random screening approach (Table 3), plant extracts, enriched fractions, or isolated compounds are randomly selected on the basis of their availability. In the context of plant-based drug discovery, this approach might be highly advantageous when applied with samples originating from regions of high biodiversity and endemism, as the chemical diversity of natural products can reflect the biodiversity of their source organisms (Barbosa et al., 2012; Henrich and Beutler, 2013). The random selection of test material has the potential to result in the identification of unexpected bioactivities that could not have been predicted based on the existing knowledge. However, the used pharmacological assays often have a small- or medium-throughput, and the starting test samples (extracts, fractions, or pure compounds) are often available only in small amounts, limiting the number of bioassays in which they can be tested. Therefore, as alternative to random testing intrinsically suffering from a low hit-rate, a variety of knowledge-based strategies can be applied to increase the probability for the identification of relevant bioactive compounds out of a smaller number of test samples with the use of a limited number of carefully selected pharmacological assays.

The classical knowledge-based approach is the ethnopharmacological approach (Table 3), where the traditional medicinal use of plants constitutes the basis for the selection of the test material and the pharmacological assay. Ethnopharmacology involves the observation, description, and experimental investigation of traditionally used drugs and their bioactivities. It represents a transdisciplinary concept based on botany, chemistry, biochemistry, and pharmacology, that involves many disciplines beyond natural science, such as anthropology, archeology, history, and linguistics (Fabricant and Farnsworth, 2001; Heinrich, 2010a; Leonti, 2011). Some prominent examples of approved drugs that were initially discovered by the use of ethnopharmacological data are: khellin from *Ammi visnaga* (L.) Lam. that served as lead compound for the development of chromoglicic acid, the sodium salt of which is used as mast cell stabilizer in allergy and asthma; galegine from *Galega officinalis* L. that was the template for the synthesis of metformin and triggered the subsequent development of biguanidine-type antidiabetic drugs; papaverine from *Papaver somniferum* L. which was the basis for the development of the antihypertensive drug verapamil; quinine from the bark of Peruvian *Cinchona* species that was used to treat malaria and inspired the synthesis of chloroquine and mefloquine which largely replaced quinine in the mid of the 20th century (Cragg and Newman, 2013; Fabricant and Farnsworth, 2001); the antimalarial drug artemisinin that has been isolated from the TCM herb *A. annua* L. in 1971 and led to the development of derivatives, such as sodium artensunate or artemether, that are nowadays widely used to treat malaria (Klayman, 1985; White, 2008).

In the well-established traditional medical systems, such as TCM or Ayurveda, ethnopharmacological knowledge is comparably easily accessible, as these systems possess an established body of written knowledge and theory that has often been revised throughout the centuries and is still in use today. In medical systems based on herbalism, folklore, or shamanism, however, no written documents exist, and the herbal formulations used are often kept secret by the practitioners, making the information more difficult to access (Brusotti et al., 2014; Fabricant and Farnsworth, 2001). Depending on the herbs to be studied, information can be acquired from different sources, including books on medical botany (e.g., Lewis, 2003), herbals (e.g., Adams et al., 2012), review articles on medicinal plants used in a certain geographic region or by an ethnic culture (e.g., Gairola et al., 2014), field work (e.g., Kunwar et al., 2009), and computer databases (Leonti, 2011; Ningthoujam et al., 2012).

A system particularly amenable to ethnopharmacological studies is TCM. In contrast to other traditional medical systems, TCM has always been regarded as a science, and it has been taught at medical schools for more than 2000 years. The first textbook fully devoted to the description of herbal drugs is the *Shen-nung-pen-ts'ao ching* (Shen Nung's Classic of Pharmaceutics). Beginning with this compendium, the first version of which was probably written down during the later Han period (25–220 AD), the literature of Chinese Materia Medica developed by continuous addition of new drugs as well as re-evaluation and addition of new indications for existing herbs during the centuries (Bauer, 1994; Unschuld, 1986; Zhu, 1998). The fact that TCM always possessed a scientific status makes it an extremely valuable source for acquisition of ethnopharmacological data, as the development in the use of medicinal plants can be readily traced back in history by studying the ancient textbooks.

The use of the ethnopharmacology-based approach, however, is associated with multiple challenges:

(1) Herbs that have been selected as study candidates based on ethnopharmacological data require not just detailed knowledge about their habitat, abundance, correct botanical authentication, whether they are threatened or endangered, and which permits are necessary in order to collect and investigate them (David et al., 2015; Fabricant and Farnsworth, 2001), but might also provoke occasions of legal right-claims from the country of origin or from ethnical groups in which the traditional knowledge was originally generated. In this context, also access and benefit sharing issues determined in the United Nation's Convention on Biological Diversity, and in the Nagoya Protocol need to be respected (see Section 2.2). These restrictions make collection of plants on an ethnopharmacological basis more tedious and time-consuming than a mere random collection, which is regarded more feasible for the common practices of pharmaceutical industry (Fabricant and Farnsworth, 2001).
(2) Some traditional systems, such as TCM and Ayurveda, involve the application of sophisticated multicomponent mixtures. The complexity of these formulations and possible synergistic effects heavily complicate the identification of active principles. On the other hand, this combinatorial approach might provide new perspectives in the treatment of multifactorial diseases, such as dementia, that might be better addressed by a multitarget-oriented, combinatorial approach (Kong et al., 2009).
(3) The definitions of health and disease in traditional medicine often widely deviate from the Western reductionist approach that is mainly based on anatomy, physiology, and cell and molecular biology. For example, the theory of TCM has been strongly influenced by Chinese philosophy, like the theory of *Yin* and *Yang*, emphasizing the balance of functional systems, and the theory of the Five Phases (*Wu Shing*) (fire, water, metal, wood, and earth), that are connected to five functional areas in the body (liver, heart, lung, kidney, and spleen and stomach) (Cheng, 2000; Uzuner et al., 2012). Such discrepancies to Western terminology often complicate the correct interpretation of ethnopharmacological data. Moreover, the holistic, personalized approach of these medical systems is difficult to assess by many of the bioassay systems currently used to prove pharmacological activity. However, the emerging omics- and systems biology-based technologies might be better suited to address these issues, due to their more holistic orientation (Guo et al., 2014; Ngo et al., 2013).

Next to the ethnopharmacological approach, another possibility for selection of plant material for the pharmacological testing is the chemosystematic or phylogenetic approach, making use of chemotaxonomic knowledge and molecular phylogenetic data in order to select plant species from genera or families known to produce compounds or compound classes associated with a certain bioactivity or therapeutic potential in a more targeted manner (Barbosa et al., 2012). Combined phylogenetic and phytochemical studies have shown that there is a strong phylogenetic signal in the distribution of secondary metabolites in the plant kingdom that can be exploited in the search for novel natural products (Saslis-Lagoudakis et al., 2011, 2012). As an example, Rønsted et al. and Larsen et al. used a phylogenetic approach to select the most promising target plants from the genus *Narcissus* and from the Amaryllidaceae tribe *Galantheae* for discovery of further acetylcholinesterase inhibitory alkaloids (Larsen et al., 2010; Rønsted et al., 2008). The combination of phylogenetic information with traditional ethnobotanical knowledge constitutes the emerging field of “phylogenetic ethnobotany” (Saslis-Lagoudakis et al., 2011). The basic assumption of this approach is that medicinal properties are not randomly distributed throughout the plant kingdom, but that some plant taxa are represented by more medicinal plants than others, and that selection of species from these “hot” taxa will lead to higher success rates in drug discovery. Particularly the exploration of cross-cultural ethnomedical patterns within a phylogenetic framework is regarded as a very powerful tool for identification of highly promising plant groups, when phylogenetically related plant species from very distant regions are found to be used for medical conditions in the same therapeutic areas (Saslis-Lagoudakis et al., 2011, 2012).

The ecological approach to select plant material is based on the observation of interactions between organisms and their environment that might lead to the production of bioactive natural compounds. The hypothesis underlying this approach is that secondary metabolites, e.g., in plant species, possess ecological functions that may have also therapeutic potential for humans. For example, metabolites involved in plant defense against microbial pathogens may be useful as antimicrobials in humans, or secondary products defending a plant against herbivores through neurotoxic activity could have beneficial effects in humans due to a putative central nervous system activity (Barbosa et al., 2012). This hypothesis might be justified due to the fact that a high proportion of biochemical architecture is common to all living organisms; considering this, it seems reasonable that secondary metabolites from organisms as distant as plants, fungi and bacteria are all able to interact with the macromolecules of the human body (Caporale, 1995). In a subtype of this approach, those plants are selected as potentially active candidates that are ingested by animals for putative self-medication purposes and for reconstitution of physiological homeostasis, e.g. in order to alleviate microbial or parasitic infection, to enhance reproduction rates, to moderate thermoregulation, to avoid predation, and to increase alertness. This concept is also referred to as zoopharmacognosy (Barbosa et al., 2012; Forbey et al., 2009; Huffman, 2003). For example, compounds with antimalarial and antiprotozoal activity could be isolated from plant species that were ingested by chimpanzees and baboons in the wild in unusual feeding behavior, supposedly in order to control intestinal parasite infection (Krief et al., 2004; Obbo et al., 2013).

Computational methods are another very powerful knowledge-based approach that helps to select plant material or natural products with a high likelihood for biological activity. These methods can also aid with the rationalization of biological activity of natural products. *In silico* simulations can be used to propose protein ligand binding characteristics for molecular structures, e.g., known constituents of a plant material. Compounds that perform well in *in silico* predictions can be used as promising starting materials for experimental work. Activity predictions using virtual screening have intriguing success rates (Hein et al., 2010), and can be conducted with a wide variety of different computational methods (Rollinger et al., 2008). *In silico* studies can focus on the main constituents of herbal remedies (Rollinger et al., 2009) as well as on any natural compounds with relevant biological effects directly retrieved from the literature. The required availability of structurally and stereochemically well-defined compounds and the approach-inherent incapability to find novel compounds, constitute the limitations of virtual screening studies. Molecular descriptors for a compound can be calculated from its 2D or 3D structure. Simple examples for such descriptors are the number of rotatable bonds or hydrogen bond acceptors. These descriptors can then be compared to datasets of active compounds to recognize correlations and establish quantitative structure activity relationship (QSAR) models to preselect compounds with a higher likelihood of activity on a specific target. In a study by Gavernet et al., for example, QSAR models were used to screen for new potential anticonvulsants in a natural products database containing 10,900 molecules. Using a series of computational filters, they proposed four hit compounds, one of which was experimentally evaluated and confirmed as active (Gavernet et al., 2008). When applying this method to plant constituents, however, it is important to stress that natural products often differ from typical synthetic pharmacologically active molecules (e.g., size, number of aromatic rings, flexibility), that are mainly found in bioactivity databases, such as CHEMBL (Gaulton et al., 2012) or PubChem (Li et al., 2010). Consequently, it is necessary to carefully evaluate datasets for compatibility (Feher and Schmidt, 2003).

**Fig. 2:**
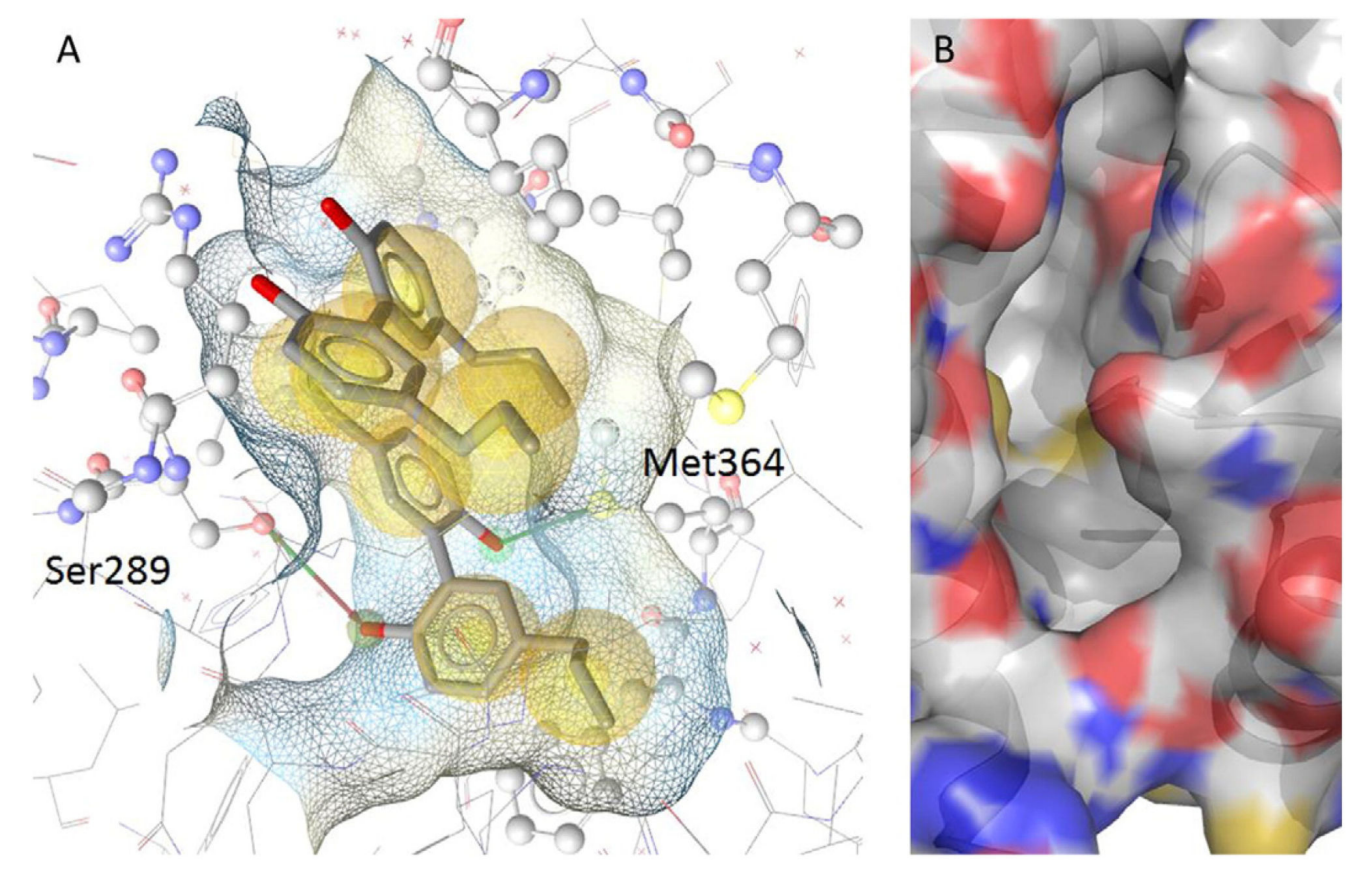
(A) Two molecules of magnolol concomitantly occupying the binding site of PPARγ (PDB 3R5N) are shown, with the chemical interaction pattern that defines the activity of the molecules depicted. Yellow spheres represent hydrophobic interactions, red and green arrows mark hydrogen bond acceptor and donor atoms. This interaction pattern may be converted into a structure-based pharmacophore model and used for virtual screening. (B) The empty binding pocket of PPARγ is shown, which can be used in docking simulations to place new molecules into the binding site and to calculate the binding free energy of the ligand.

Pharmacophore-based virtual screening constitutes another highly successful computational method. A pharmacophore model is a 3D arrangement of physicochemical features (e.g., hydrogen bond donor/acceptor, hydrophobic area, aromatic ring) that represents the key interactions between a ligand molecule and its target protein. As an example, the chemical interaction pattern that defines the interaction of magnolol with the binding site of PPARγ (PDB 3R5N) is presented in Fig. 2A [for more details about the significance of this example the reader is referred to (Zhang et al., 2011) and (Fakhrudin et al., 2010)]. 3D-multiconformational compound libraries can be screened against a pharmacophore model to retrieve molecules that map the pharmacophore features and consequently have a high likelihood of being active on the target. Depending on the target, this method can achieve success rates between 2 and 30% (Hein et al., 2010). If pharmacophore models are available for a range of targets, parallel virtual screening can be used for so-called “target fishing” (Schuster, 2010). This method can be highly valuable for target identification if a general activity of an extract or pure compound is known. The structure can be screened against a set of models for multiple targets to reduce the experimental work identifying the molecular target(s) related to the bioactivity (Duwensee et al., 2011; Steindl et al., 2006; Wolber and Rollinger, 2013).

A third computational method, molecular docking, is widely used to elucidate the mechanism of action and rationalize structure activity relationships of natural products. The aim of docking is to accurately predict the positioning of a ligand within a protein binding pocket and to estimate the strength of the binding with a docking score (Waszkowycz et al., 2011). As an example, Fig. 2B visualizes the empty binding pocket of PPARγ, which can be used in docking simulations to place new molecules into the binding site and to calculate the binding free energy of the ligand. If the 3D structure of a protein is available, either from X-ray crystallography, NMR data, or through homology modeling, then ligand molecules can be computationally positioned directly in the binding pocket to analyze their putative target-ligand interactions and thus identify the crucial binding features of the molecule. Docking can also be employed in large scale virtual screenings, where a molecule is docked into a series of targets and a suitable docking score is used to compare the results to identify the best ranked matches. Docking has been widely employed to rationalize the structure–activity relationship of natural products. This was demonstrated for example recently with constituents of *Carthamus tinctorius* L., which showed different activities on indoxyl indoleamine 2,3-dioxygenase (Temml et al., 2013).

Computational methods also provide the means to discover previously undescribed binding sites on known protein structures. Pocket finders detect solvent-accessible cavities in the protein surface that can indicate potential ligand binding sites. These sites can then be analyzed computationally. In a study conducted by Hanke et al., for example, this approach was used to identify binding sites for a series of aminothiazole-featured pirinixic acid derivatives, which showed dual activity on 5-lipoxygenase and microsomal prostaglandin E_2_ synthase-1, and new potential binding sites for both enzymes were identified (Hanke et al., 2013).

Whereby *in silico* methods represent valuable filter tools in the search of new activities for natural products, they can also be employed to predict ADME/T properties (Kaserer et al., 2014) or to find new activities for already approved drugs (drug repurposing) (Steindl et al., 2007).

Several virtual libraries comprising collections of natural products have been reported. For example, the DIOS database is a collection of 9676 literature-derived compounds from plants described by Pedanius Dioscorides in his fundamental encyclopedia *De Materia Medica* (1st century AD), which is widely regarded as the precursor to all modern pharmacopeias. The Natural Product Database (NPD) is a more general database, that collects more than 122,700 compounds from natural sources (Rollinger et al., 2004). In the CHM database, 10,216 compounds from traditional medicines are collected for virtual screening (Fakhrudin et al., 2010). The comprehensive Dictionary of Natural Products (DNP) is a commercially available subset library of the Chapman & Hall/CRC Chemical Database. In 2012, ZINC announced the availability of the ZINC Natural Product Like database. In the ZINC, also commercially available and literature-derived natural compound databases are provided for download and virtual screening (Znplike; http://zinc.docking.org/browse/subsets/special).

### 3.2. Considerations regarding the choice of bioassays

Drug discovery from plants requires a multidisciplinary approach in which the success is largely dependent on a well-chosen set of *in vitro* and *in vivo* assays. The choice of the bioassays, first and foremost determined by the study objectives, should optimally combine simplicity with good sensitivity and reproducibility.

**Fig. 3:**
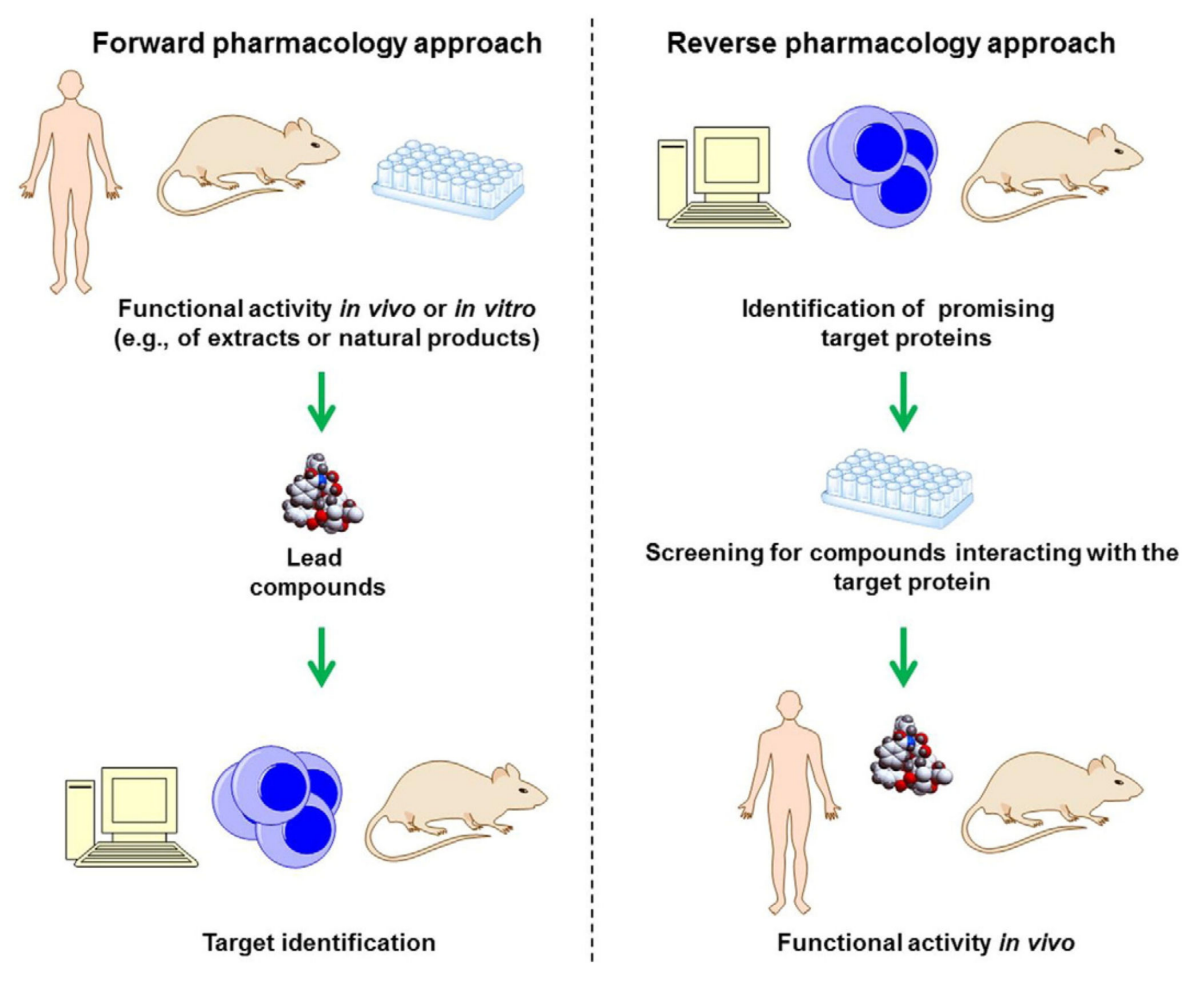
The forward pharmacology and reverse pharmacology approaches in natural product-based drug discovery.

Historically, the investigation of plant-derived substances was based on a forward pharmacology approach using *in vivo* animal tests, organ or tissue models, or bacterial preparations, followed by *in vitro* investigation of mechanistic underpinnings. In the more recent past, the approach of investigating plant-derived substances changed and is now usually starting with screening of large collections of plant-derived compounds (“libraries”) against pre-characterized disease-relevant protein targets, with the aim to identify “hits”, compounds with the desired activity that are then further studied in relevant *in vivo* models with the aim to validate them (a reverse pharmacology approach). Both the forward and the reverse pharmacology approaches use an overlapping selection of bioassays but differ in the stage when the assays are applied (Fig. 3; (Lee et al., 2012; Schenone et al., 2013; Takenaka, 2001; Zheng et al., 2013)).

The forward pharmacology, also known as phenotypic drug discovery, first determines functional activity by detecting phenotypic changes in complex biological systems and then characterizes the molecular target of the active compounds. This traditional way of drug discovery was carried out mainly in the era before the Human Genome Project, and especially before the development of many of the modern molecular biology techniques.

The reverse pharmacology, also known as target-directed drug discovery, starts by identifying a promising pharmacological target against which compounds are screened and then obtained promising hit compounds are validated *in vivo*. Both unbiased (random) compound libraries as well as knowledge-based libraries (see Section 3.1) can be used with this approach. While the reverse pharmacology approach has the advantage of reduced animal testing, its disadvantage is that it often requires a huge amount of time and effort for the initial stages, without a guarantee for *in vivo* efficacy.

It must be noted that in the medicinal plant research community in the last decade the term “reverse pharmacology”, is also used sometimes to designate “bedside-to-bench” or “field to pharmacy” strategies starting with clinical efficacy data followed by *in vivo* and *in vitro* mechanistic studies (Aggarwal et al., 2011; Graz, 2013; Patwardhan and Vaidya, 2010; Vaidya, 2006). However, this terminology use is conflicting with the mainstream understanding existing in the broad drug discovery scientific community, which would see the “bedside-to-bench” strategy as a classical forward pharmacology (phenotypic drug discovery) example starting with observation of phenotypic changes at organismal level [e.g., reversal of disease symptoms in patients; (Lee et al., 2012; Schenone et al., 2013; Takenaka, 2001; Zheng et al., 2013)]. In this sense, terminology standardization is needed in order to avoid miscommunication between researchers from different scientific disciplines.

The importance of a proper selection of the initially used pharmacological assay is underlined by the fact that lack of clinical efficacy (indicative for inappropriate pre-clinical models) is among the most important reasons for failure of novel drugs during development (Kola and Landis, 2004; Schuster et al., 2005). In this section we present a brief overview of assays used to determine bioactivities of phytochemicals on the protein-, cell-, or organism level. For further information on the topic, we refer the reader to the following excellent reviews: (Agarwal et al., 2014; Butterweck and Nahrstedt, 2012; Fallarero et al., 2014; Wang et al., 2011a). Methods for activity assaying that are based on simple chemical reactions, such as some widely used methods to determine *in vitro* antioxidant properties (e.g., the 2,2-diphenyl-1-picrylhydrazyl (DPPH) radical scavenging assay), have been reviewed elsewhere (Gulcin, 2012; Moon and Shibamoto, 2009) and will not be discussed in detail here.

Assays to detect bioactivities of natural products comprise *in vitro* models with purified proteins, cell-based target-oriented or phenotypic assays, models with isolated tissues or organs, and *in vivo* preclinical animal models. These methods mainly differ in their complexity and throughput capacity and exhibit some advantages and disadvantages, as covered in Table 4.

Classical protein-based *in vitro* assays rely on the measurement of the functional activity of the investigated target protein in the presence of a test compound, or of the physical interaction of the test compound with the target protein. This class of assays can be usually performed in any general-purpose laboratory without the need for cell culture or animal facilities and are well suited for HTS. A recent evaluation of the drugs that were approved by the FDA during the past three decades (Rask-Andersen et al., 2011) revealed that the biggest group of protein targets of approved drugs so far are receptors (193 proteins targeted by 563 approved drugs), followed by enzymes (124 proteins targeted by 234 drugs), transporter proteins (67 proteins targeted by 181 drugs), and other types of targets (51 types, targeted by 84 drugs). The best represented target classes were the G-protein coupled receptors (GPCRs) in the receptor group, the hydrolases in the enzyme group, and the voltage-gated ion channels in the transporter protein group (Imming et al., 2006; Rask-Andersen et al., 2011).

Observed inhibition in *in vitro* protein assays often reflects binding of the test compound to the active center of the target protein, thereby blocking it. In other cases, the assay might be designed to reflect the inhibition of a protein–protein interaction required for the functional activity, or protein activation induced upon compound binding. While these assays inherently provide a “mechanism of activity” of the identi-fied inhibitor or agonist, they cannot guarantee its functionality in more complex biological systems such as cells and multicellular organisms. As a result, many promising “hits” fail when further tested in cell-based *in vitro* assays or *in vivo* animal experiments. Peculiarly, in spite of the current focus of the pharmaceutical industry on target-oriented *in vitro* screening, analysis of the first-in-class new molecular entities (NMEs) approved by the FDA between 1999 and 2008 revealed that the majority of them was first discovered using phenotypic assays (28 compounds compared to 17 identified by target-based approaches). The surprising result of this analysis led the authors to postulate that a target-centric approach for first-in-class drugs may significantly contribute to the current high attrition rates and low productivity in pharmaceutical research and development (Swinney and Anthony, 2011). In spite of this, it should be noted that assays with purified proteins have been extensively and on many occasions very successfully employed in many different drug discovery programs e.g., in the identification of selective inhibitors of various GPCRs and kinases (Alkhalfioui et al., 2009; Cohen and Alessi, 2013; Zaman et al., 2003).

Assays with cultured mammalian cells are a relatively simple and inexpensive alternative to *in vivo* assays for the initial assessment of pharmacological activity. They are also applicable for bioactivity-guided isolation of natural products from plant extracts. Compared to protein-based *in vitro* assays, they provide bioactivity information at the cellular level. Assays with cultured mammalian cells are mostly used in academic institutions for low to medium throughput screening. However, this type of assays is also amendable for up-scaling to screen very large compound libraries, and in this form is still widely used primarily, but not exclusively (Larsen et al., 2014), by the pharmaceutical industry.

The broad spectrum of available cell-based assays requires a careful and meaningful selection of the assay design, depending on the aim of the investigation and the intended throughput. The type of process that is studied often dictates the selection of the cell type that needs to be used. For example, endothelial cells are selected to study angiogenesis; epithelial cells might be used for dermatological research. Furthermore, cell-based assays might be target-oriented or phenotypic (Table 4). Target-oriented assays are providing information on the interference of investigated compounds with the function of a specific protein or pathway [e.g., (Atanasov et al., 2013b; Orlikova et al., 2013; Reuter et al., 2009)], whereby phenotypic models are providing information on a changed cellular phenotype with a complex regulation [e.g., cell proliferation (Kurin et al., 2012; Schwaiberger et al., 2010)]. Cell-based assays might be performed using primary mammalian cells or cell lines. Although immortalized cell lines are easier to maintain in culture, often results obtained with them are less relevant, because immortalization and prolonged *in vitro* maintenance allows accumulation of mutations and phenotypic changes. Used cells or cell lines can also be derived from genetically modified transgenic animals. Other approaches resort to engineering of cells *in vitro* to stably overexpress, stably down-regulate, or knockout a gene product of interest (Ketting, 2011; Wang et al., 2014c). The spectrum of cell-based assays has been widened by engineering of cells enabled to act as reporters (e.g., luciferase- or fluorescence-based) of a specific intracellular response influenced by the tested substance (Baird et al., 2014; Fakhrudin et al., 2014). Aside of the use of reporter systems, a variety of phenotypic parameters have been successfully selected as readout, e.g., changes in cell morphology, cell adhesion, cell proliferation, migration, differentiation status, metabolic status, redox status, cell apoptosis or senescence (Atanasov et al., 2013b; Blazevic et al., 2014; Gaascht et al., 2014; Zheng et al., 2013). Phenotypic cell-based assays can be used to verify the activity of compounds identified by protein-based *in vitro* assays at the cellular level. Moreover, cell-based phenotypic models might also be used to study the underlying molecular mechanisms of certain biological effects, possibly leading to the discovery of new target molecules or pathways affecting the respective phenotype. For this purpose, changes in intracellular signaling or gene expression are often characterized by techniques such as immuno-cytochemistry, FACS-analysis, real-time PCR, Western blotting, immunoprecipitation, or “omics”-techniques (e.g., genomics-, transcriptomics-, proteomics- and metabolomics-techniques) for characterization of global changes in gene expression or metabolite quantities (Ladurner et al., 2012; Lee and Bogyo, 2013; Schenone et al., 2013; Schreiner et al., 2011; Ziegler et al., 2013).

Besides mammalian cells, also yeasts have been employed for the establishment of whole-cell phenotypic assays with applications in drug discovery, e.g., for high throughput functional screening based on the activation of caspases and other proteases involved in cell death and inflammation (Hayashi et al., 2009), as a screening tool for pharmacological modulation of GPCRs (Minic et al., 2005), but also to understand the mechanism of action of drugs or to identify novel drug targets and target pathways (Hoon et al., 2008).

A special set of assays that stay on the interface of *in vitro* and *in vivo* models encompasses *in situ* and *ex vivo* methods with isolated tissues or organs (Luo et al., 2013; Teicher, 2009). They show the advantage of usually resembling the *in vivo* situation more closely than the *in vitro* tests, but also the disadvantages of lower throughput, more difficult resupply, ethical concerns related to the use of animals, and the short half-life of the isolated tissues and organs. Into this group of methods also falls the recently described technology *Ex Vivo* Metrics^™^ that uses intact human organs ethically donated for research (Curtis et al., 2008). While most of the mentioned limitations of *ex vivo* models are applicable also to the *Ex Vivo* Metrics^™^ technology, its strong advantage is that it eliminates potential species differences and offers the closest applicable biological system in terms of emulating human exposure to drugs prior to clinical trials (Curtis et al., 2008).

In summary, even though the relevance of *in vitro* assays is limited due to the inability to provide information on factors influencing the activity of compounds *in vivo* (such as ADME/T), *in vitro* assays still represent very important tools to identify and characterize bioactive compounds.

Following the reverse pharmacology approach (Fig. 3), compounds identified with a good activity *in vitro* need to be tested *in vivo* in suitable animal models that can provide basic pharmacological and toxicological data prior to subsequent human clinical trials.

Traditionally, in part due to the reasonably high homology and similarity between mammalian genomes and physiology, and to the relatively short reproductive cycle, mouse and rat models were used for the assessment of the activity of plant extracts or isolated natural products (Butterweck and Nahrstedt, 2012). Such animal models are still crucial for drug evaluation and validation, as they provide the investigator with an integrated response encompassing efficacy, bioavailability, side effects, and toxicity (ADME/T parameters) of the drug in a whole organism, and are standardly used for pharmacokinetic and safety studies that are a pre-requisite for human clinical trials (Alqahtani et al., 2013; Eddershaw et al., 2000; Parasuraman, 2011; Zhang et al., 2012). Non-rodent mammalian species such as rabbits, dogs, swine, and monkeys are also widely used in pharmacological safety and pharmacokinetic studies (Parasuraman, 2011; Pellegatti, 2013; Swindle et al., 2012; van der Laan et al., 2010; Zhang et al., 2012). Although such large animal models are associated with limitations, such as higher price and more pronounced ethical considerations, their use is still widely practiced, especially by the pharmaceutical industry, since the regulatory guidelines of FDA, European EMA, and other similar international and regional authorities usually require safety testing in at least two mammalian species, including one non-rodent species, prior to human trials authorization (Parasuraman, 2011; van der Laan et al., 2010). Indeed, rodent and human proteins are sometimes not similarly sensitive toward bioactive compounds (Huang et al., 2004; Kervinen et al., 2010), and therefore a higher confidence for safety and efficacy can be obtained by using several mammalian species, or genetically engineered “humanized” animal models if available (Foster et al., 2014; Scheer and Roland Wolf, 2013). In this context, an additional factor contributing to the broad use of rodents as first-line *in vivo* models for pharmacological testing is the availability and the established methods for de novo generation of genetically modified mice (with a gene-knockout, downregulation (knockdown), or overexpression of a protein of interest), as well as genetically modified rats, which are providing a set of additional approaches to study pharmacological effects. Such genetically modified rodent models are successfully applied for the verification of *in vivo* efficacy prior to human trials, for the discovery of new pharmacological targets, as well as to assist the elucidation of pharmacological mechanisms of action (Foster et al., 2014; Zambrowicz and Sands, 2003; Zhang et al., 2012). The generation of genetically modified animal models has dramatically evolved recently by the development of new TALEN- and CRISPR/Cas9-mediated techniques for genome editing (Harrison et al., 2014; Miller et al., 2011; Wang et al., 2013a).

General limitations associated with the use of rodents or other mammals for scientific purposes are higher costs, incompatibility with modern HTS, and growing ethical concerns [which led for example to the recent introduction of the Directive No. 2010/63/EU of the European Parliament promoting the “3R principle” (Replace, Reduce and Refine) for the use of animal experiments]. In spite of these limitations, mouse and rat remain the most widely used and still indispensable species in the drug discovery process. When using rodent animal models for *in vivo* verification of efficacy, a number of parameters need to be carefully considered, such as the route of administration of the test substances, the dosage to be applied, the experimental readout (that needs to be specific and sensitive), and the employment of an established positive control, if available (Vogel, 2002).

Non-mammalian animal models, like the nematode *Caenorhabditis elegans* and zebrafish (*Danio rerio*) have the advantage that they offer the opportunity for medium- to high-throughput screening in intact living organisms. Their wide-spread use has been promoted by elaborating the instrumentation for a fully automated medium- to high-throughput screening with nematodes (O'Reilly et al., 2014; Pulak, 2006) as well as with zebrafish (Gibert et al., 2013; Makky et al., 2008). The applicability of these species as disease models has been additionally expanded by recent implementation of the techniques of gene editing in both nematodes (Wood et al., 2011) and zebrafish (Huang et al., 2011), allowing for rapid and low-cost genetic engineering. To give one example for the potency of the zebrafish screening model in the context on plant-derived natural product pharmacology, zebrafish has recently been successfully used in a sterile tissue injury model of inflammation resolution for screening of 2000 compounds including approved drugs, natural products, and defined pathway inhibitors. This study led to the identification of tanshinone IIA (derived from the Chinese medicinal herb *Salvia miltiorrhiza* Bunge), which potently induces resolution of inflammation *in vivo* (Robertson et al., 2014). Importantly, the effects found in zebrafish-neutrophils were also observed in human neutrophils, supporting the applicability of this model to screen for drugs aimed to be used in humans.

### 3.3. Approaches for the identification of active plant constituents

A very common approach is to start pharmacological testing with crude plant extracts and subsequently to isolate and characterize the constituents responsible for the activity of the extract (Koehn and Carter, 2005). Preparation of plant extracts is relatively simple and low in cost and time investment, but to assure good data reproducibility, it is crucial to carefully document identity, history, and processing of the plant material (see Section 2.2). The extraction method can strongly influence the chemical composition, and thus the biological activity of the extract. Therefore, the selection of the extraction method, including the solvent, needs to be carefully considered. If the starting material originates from plants used in traditional medicine, it is often recommended to perform the extraction in a way that mimics the preparation of the traditional herbal drug, although this is often difficult, e.g. when fresh plant material is used for the traditional application (Heinrich, 2010a). If the structural classes of the target compounds are known, the extraction and fractionation procedures can be optimized to maximize the yield. However, often the chemistry of the bioactive compounds in the investigated plant is not known, and in such cases it is advantageous to extract a broad spectrum of compound classes. Often, the initial crude extracts are prepared with 70% aqueous methanol or ethanol, since these solvents have proven in practice to be suitable to extract a broad range of bioactive molecules. An often used alternative to the extraction with one solvent is the preparation of multiple crude extracts by subsequent extraction of the plant material with solvents of increasing polarity (e.g., *n*-hexane, dichloromethane, and methanol). The major advantage of this approach is that the generated multiple crude extracts contain a smaller variety of compounds, and the major disadvantage is that more crude extracts need to be biologically assayed (Liu, 2008).

Crude plant extracts exhibiting biological activity are subjected to iterative bioactivity-guided fractionation cycles until the respective pure bioactive compounds are identified [Table 5; (Liu, 2008)]. Since the fractionation and bioactivity evaluation of the generated fractions are very laborious steps, it is critical to obtain reliable bioactivity data with the crude plant extracts, optimally in independent complementary bioassays. Typically, liquid–liquid extraction (Huang et al., 2007; Pantev et al., 2006) using organic solvents of different polarity (e.g., ethyl acetate, *n*-butanol, and chloroform) in water is conducted as a first fractionation step. In order to guide the isolation process, fractions obtained from bioactive crude extracts are subjected to biological investigation (best in direct comparison with the crude extract). Following each fractionation step, inactive fractions are dropped, and active fractions are subjected to further separation, most often by column chromatography. If the testing is done at the same (w/v) concentration, the bioactivity of fractions containing the active constituents is expected to increase in each following fractionation cycle, compared to the crude extract, because the relative abundance of the active constituents in these fractions is increasing. Most often, several chromatographic steps are necessary to obtain the pure active compounds, which are then identified using different spectroscopic techniques (Liu, 2008).

The above described workflow of traditional bioactivity-guided isolation is time-consuming, labor- and cost-intensive, but nonetheless can result in the successful identification of relevant constituents with novel bioactivities (Baumgartner et al., 2010; Heiss et al., 2014). However, it also might lead to known compounds with already known bioactivities (Vogl et al., 2013; Xie et al., 2014). Moreover, since plant extracts represent complex mixtures comprising a multitude of different components (active, partially active, as well as many inactive compounds) (Heinrich, 2010b), there are often interactions between constituents. Therefore, a high activity of a crude extract may result from the presence of many weakly active compounds which act additively or synergistically (Junio et al., 2011; Kurin et al., 2012; Wagner and Ulrich-Merzenich, 2009; Zimmermann et al., 2007). In such a scenario, fractionation will disrupt these interactions and thus result in a decrease of the activity. It is also possible to miss a promising bioactivity if the concentration of the bioactive compound in the crude extract is too low. Furthermore, a promising bioactivity can also be masked or diluted by highly abundant constituents in the extract. Ubiquitous bulk substances, such as chlorophyll and polyphenols (e.g., tannins) may interfere with biological assays and generate false positive or false negative results. Whereas chlorophyll was shown to interact with fatty acids and to possess anti-oxidative activity (Cho et al., 2000; Park et al., 2007; Potterat and Hamburger, 2006), tannins are known to form complexes with polysaccharides, proteins, and metal ions, to interfere with cell-based activity assays, and also to display anti-oxidative effects (Cos et al., 2006; Tan et al., 1991; Wei et al., 2011b; Zhang et al., 2010).

An emerging alternative to bioactivity-guided fractionation is the metabolic profiling approach (Table 5). It correlates the chemical profile and the bioactivity pattern of plant extracts, to guide the isolation of compounds, and to identify new or already known bioactive constituents at an early stage (dereplication) (Tawfike et al., 2013; Wolfender et al., 2014). The goal of metabolomics in general is to analyze all secondary metabolites in a sample qualitatively and quantitatively. Compared with other “omics” techniques such as genomics or proteomics, plant metabolomics is considered to be the most function-oriented approach to identify the biochemical status of the plant, since metabolites are the end products of cellular regulatory processes. Therefore, their levels can be viewed as the ultimate response of biological systems to genetic or environmental changes (Yuliana et al., 2011). The identification and quantification of secondary metabolites in plant extracts in general is highly challenging (Ebada et al., 2008; Kjer et al., 2010) due to the diversity of the chemical and physical properties and the wide range of concentrations of the analytes (Dunn et al., 2005). Therefore, highly sensitive and reproducible analytical methods are required. Usually (ultra) high performance liquid chromatography [(U)HPLC] or gas chromatography (GC) coupled to (high resolution) mass spectrometry [(HR)-MS], and nuclear magnetic resonance (NMR) spectroscopy, are applied. The analytical data need to be pre-processed prior to statistical analysis. Different open-source and commercial software packages are available for this task (Creek et al., 2011; Hartler et al., 2011; Jankevics et al., 2012; Kamleh et al., 2008; Kawaguchi et al., 2010; Pluskal et al., 2010; Smith et al., 2006). Preprocessed analytical data from a high number of samples showing distinct pharmacological activities can then be subjected to multivariate data analysis. As a first step, usually unsupervised methods such as principal component analysis (PCA) are used in order to examine the distribution of the samples (e.g. active vs. inactive ones) and to identify outliers (Wolfender et al., 2013). In a second step, correlation of analytical and pharmacological data by supervised methods like partial least square regression modeling discriminant analysis (PLS-DA) or orthogonal projection to latent structures discriminant analysis (OPLS-DA) enables the identification of features that are highly correlated with the biological activity. This allows the targeted isolation of novel putatively bioactive compounds, as well as the dereplication of already known ones (Cardoso-Taketa et al., 2008; Maree et al., 2014; Wang et al., 2013b). Therefore, this approach can shorten the bioactivity-guided isolation process by enabling the detection of the bioactive compounds at an early stage and therefore preventing unnecessary efforts, costs, and time of working steps (Tawfike et al., 2013). Wolfender et al. have recently extensively reviewed the still existing challenges underlying this approach (Wolfender et al., 2014). The main obstacle is the comprehensive detection of the metabolites occurring in complex natural extracts by highly reproducible methods, that often requires the application of several extraction methods (Choi and Verpoorte, 2014) and of several complementary sophisticated analytical platforms, involving expensive high-end analytical instruments (Wolfender et al., 2014). Also pre-processing and data mining are critical steps due to the high complexity of the analyzed samples and the wide variety of existing software packages that are based on different algorithms and therefore can extract different features. Dereplication and unambiguous identification of metabolites is particularly problematic in case of LC–MS data, since different instrument types generate different ion species and fragmentation patterns, thus complicating the setup of generic databases (Wolfender et al., 2013).

The bioactivity-guided isolation and the metabolic profiling approaches focus on the isolation and identification of active principles from identified active extracts. On the contrary, the direct phytochemical isolation approach focuses on the full chemical characterization of a plant extract. By using dereplication tools (e.g., LC–MS), special emphasis is put on the isolation of novel compounds. The isolated compounds are subjected directly to biological testing. Compounds isolated with this approach might also be assembled in natural product libraries, for example the National Cancer Institute's natural product library (Liu et al., 2013), that can be used for different bioactivity tests, including HTS. Starting the bioactivity screening with pure compounds has many advantages including better compatibility with HTS and prevention of many difficulties, which come along with extract testing as mentioned above. However, using this approach, time and effort need to be invested in advance for the isolation of the natural compounds without any guarantee that the respective compounds will finally have a relevant bioactivity. Next to the possibility for HTS with libraries composed of randomly selected natural products, a variety of knowledge-based strategies can be applied for the assembly of focused natural product libraries including just natural products with certain characteristics (see Section 3.1). In this respect, a good prioritization approach might be to use chemography to navigate in chemical space (Oprea and Gottfries, 2001). Chemical space is the entity of all possible molecules, comprising an estimated number of more than 10^60^ compounds only when small carbon-based molecules are considered and a significantly higher number when adding more complex bioactive molecules (Bohlin et al., 2010; Larsson et al., 2007). The region of chemical space relevant for natural product-based drug discovery is termed “biologically relevant chemical space”. The limits of this multidimensional sub-volume are defined by properties and boundaries that allow binding interactions between biological molecules [primary and secondary metabolites, polypeptides, enzymes, RNA, DNA; (Bohlin et al., 2010; Lipinski and Hopkins, 2004; Oprea and Gottfries, 2001)]. In order to enable the exploration of biologically relevant space, ChemGPS-NP, a prediction model tuned for the exploration of the regions of chemical space most likely to enclose compounds with biologically relevant functions and activities, has been developed [http://chemgps.bmc.uu.se; (Larsson et al., 2007; Rosen et al., 2009)]. This online tool can be applied for compound selection and prioritization, property description and interpretation, cluster analyses and neighborhood mapping as well as comparison and characterization of large compound datasets (Rosen et al., 2009).

Starting the screening with enriched fractions, containing a smaller number of compounds in comparison with crude extracts, is an approach that is in-between the other two. There is less preliminary work required in comparison with the approach directly starting with pure compounds. In addition, less subsequent work is required to isolate and identify the bioactive compounds compared to the approach starting with crude extracts. However, starting the screening with enriched fractions is based on a higher number of starting test samples since multiple fractions are generated from a single plant extract. In cases when the pharmacological assays allow HTS, this approach is advantageous, since many HTS approaches that do not work well with plant extracts due to interferences with ubiquitous perturbing compounds, could be successfully applied to enriched fractions (Eldridge et al., 2002; Hassig et al., 2014; Henrich and Beutler, 2013; Johnson et al., 2011). In addition to HTS with libraries of randomly obtained plant-derived fractions, also in this occasion different knowledge-based strategies can be applied for the assembly of focused libraries containing just fractions with certain characteristics (see Section 3.1). For example, an approach for selection of extracts and fractions based on their drug-like physicochemical properties was recently developed (Camp et al., 2012).

Since the bioactivity of a plant extract may be the result of synergistic interactions of several components, and in this case bioactivity-guided fractionation might fail, a synergy-directed fractionation strategy was recently developed (Junio et al., 2011). This approach combines bioactivity testing of the generated fractions for synergistic interactions, with MS-profiling and natural products isolation, aiming to identify synergistic interactions of extract constituents which could be missed using the traditional bioactivity-guided fractionation.

Noteworthy, in addition to the identification of single bioactive plant constituents, there is also an increasing interest in the use of standardized plant extracts as herbal medicinal products or dietary supplements. Especially if there are synergistic or additive effects, if bioactive compounds are more stable in the extract, or if the bioavailability is better, an extract might possibly have higher therapeutic efficacy compared to the isolated pure compounds. However, more research is needed in this direction (Gertsch, 2011; Liu and Wang, 2008; Liu, 2008; Schmidt et al., 2008).

Another important aspect related to the identification of plant-based bioactive molecules, is that it is now increasingly recognized that some natural products are prodrugs that need to be metabolized *in vivo* by the intestinal microorganisms or by the mammalian organism in order to yield pharmacologically active molecules (Akao et al., 2002; Atkinson et al., 2004; Butterweck and Nahrstedt, 2012; Hostanska et al., 2014; Tomas-Barberan et al., 2014). This is posing serious challenges to some of the established approaches related to bioactivity investigations of traditionally used medicinal plants, since in such occasions the effector compounds are not present in the starting plant material and bioactivity cannot be detected with many conventionally used assays and approaches [e.g., *in vitro* assays (Table 4) and direct phytochemical isolation approach (Table 5) are not applicable]. An emerging concept, termed here “metabolism-directed approach” (Table 5), is addressing these challenges by the use of *in vivo* models or *in vitro* assays specifically designed for a targeted search of bioactive plant metabolites (Actis-Goretta et al., 2012; Bowey et al., 2003; Guo et al., 2008; Qiao et al., 2012; Wang et al., 2014b; Xiao and Hogger, 2013; Yu et al., 2014).

## 4. Approaches for resupply of pharmacologically active plant compounds

Many potent plant-derived natural products such as paclitaxel, podophyllotoxin, or vinblastine share severe difficulties to meet the market demands, as their natural sources are often slow-growing or even endangered species that tend to accumulate these compounds at very low quantities over long growth periods (Miralpeix et al., 2013; Staniek et al., 2014). Natural populations of many medicinal plants have been increasingly facing pressure from mostly anthropogenic factors like environmental damage, deforestation and industrialization due to extensive population growth, fires and other natural disasters, development of land for agriculture, climate change, and last but not least, extensive collection of plant material from the wild (Arora et al., 2010). In fact, more than 20% of the world's medicinal and aromatic plant species are threatened to varying degrees (Schippmann et al., 2006). Two thirds of the estimated 50,000 medicinal plant species currently used are collected from the wild, raising concerns about issues such as diminishing populations, loss of genetic diversity, local extinctions, and habitat degradation (Canter et al., 2005). While isolation of natural products directly from the plant species in which they occur is often acceptable if small to moderate amounts of the respective compounds are needed (for example for the supply of sufficient quantities for small-scale experimental laboratory work), additional routes for resupply need to be considered once there are increased market demands for the respective natural product. In these cases natural products can be resupplied by the application of plant cell and tissue culture, heterologous production, total chemical synthesis, or semi-synthesis from isolated precursors occurring more abundantly in nature, as discussed in greater detail in the following subsections.

### 4.1. Plant cell and tissue culture

Cultivation of endangered medicinal plants under controlled conditions represents a promising protective approach (Tasheva and Kosturkova, 2013). For some of the stages which make up the process of domestication of wild-growing plant species, *in vitro* techniques can be applied, for example in selection breeding for genetic improvement or storage of plant materials (Bohr, 1997; Franz, 1991). Since the fundamental report by Haberlandt on the *in vitro* cultivation of isolated plant cells (Haberlandt, 1902), plant cell and tissue culture has developed into an important and well established discipline, with impact on both basic research and applied technologies. Out of these, the *in vitro* propagation (micropropagation) of plants and the *in vitro* culture of plant organs (typically roots) can provide means for the supply of plant material as source of natural products. Plant cell culture (i.e. the *in vitro* culture of isolated cells) has long been regarded as a convenient tool for the biotechnological production of plant compounds, but as outlined later, the practical applicability of this approach should be critically questioned.

Micropropagation is defined as the *in vitro* plant regeneration from excised plant parts through either shoot organogenesis or somatic embryogenesis (Siahsar et al., 2011). On a commercial scale it has developed into a multi-billion-dollar industry and offers important advantages over the conventional propagation of plants, like production of large numbers of genetically homogenous plants year-round in a comparatively short time, disease-free propagules, and greatly enhanced multiplication rates (Debnath et al., 2006). Micropropagation has become a standard method of plant production for many species of economic value, such as plants of agricultural, ornamental, vegetable, and forestry importance (Debnath et al., 2006; Read, 2007; Tasheva and Kosturkova, 2013). *In vitro* propagation certainly also holds significant potential for the mass production of medicinal plants and numerous protocols for micropropagation of a huge number of medicinally used species have been developed up to date (Afolayan and Adebola, 2004; Chaturvedi et al., 2007; Debnath et al., 2006; Rout et al., 2000; Sarasan et al., 2011; Tripathi and Tripathi, 2003; Wawrosch, 2010). However, the higher costs of *in vitro* techniques as compared to conventional propagation are a major disadvantage and have limited its use at a commercial level (Mehrotra et al., 2007; Pence, 2011; Sahu and Sahu, 2013; Wawrosch, 2005). Although medicinal plants are increasingly introduced into field culture (Wiesrum et al., 2006), general obstacles to the commercial cultivation of many medicinal plants include a difficulty to predict which herbal products will remain marketable (Debnath et al., 2006), economic impediments in case of small market sizes, and last but not least the fact that wildly collected material is less expensive (Lubbe and Verpoorte, 2011).

Plant cell cultures (dedifferentiated cells suspended in liquid nutrient medium) have been aptly denominated as chemical factories of secondary metabolites (Rao and Ravishankar, 2002). They offer a number of advantages over the conventional use of plants as sources of phytochemicals, like for example independence of geographical, seasonal, and environmental variations; continuous reliable production in uniform quality and yield; avoidance of pesticide and herbicide application; and comparatively short growth cycles (Bonfill et al., 2013; Debnath et al., 2006; Rao and Ravishankar, 2002). Technologies which have been developed for other cell culture systems like mammalian cells or yeast can be adapted for large scale industrial applications with plant cells (Kolewe et al., 2008). But, despite of many decades of research in the field, at present only fourteen substances or products are produced commercially from plant cell cultures (Frense, 2007; Kolewe et al., 2008), with the most prominent being paclitaxel from Taxus spp. This is due to several limitations of plant cell cultures in comparison to microbial production sources, including slow growth rates and low and variable yields of metabolites — in fact, many metabolites do not accumulate in plant cell cultures because of lacking differentiation and compartmentalization (Kirakosyan et al., 2009; Kolewe et al., 2008). While still very little is known about the plant secondary metabolites biosynthesis and its regulation, it remains questionable whether in a foreseeable time plant cell cultures will play a significant role in the industrial production of natural products. Nevertheless, in order to get a broad overview of the existing resupply approaches, some aspects of the methodology will be briefly addressed here.

Noteworthy, in many cases the formation of desired compounds in plant cell cultures can be distinctly enhanced by different measures. In this regard, one important approach is the modification of the nutrient medium with the aim of increasing biomass and, often in a subsequent step, optimizing metabolite production (Bonfill et al., 2013; Murthy et al., 2014; Weathers et al., 2010). Herein, key factors include the ionic strength of the basal medium, phosphate and nitrate level, the level and type of sugars, growth regulators, and the feeding with precursors (Murthy et al., 2014; Rao and Ravishankar, 2002). Furthermore, a number of physical factors can influence the properties of plant cell cultures. These are the inoculum density, temperature, light quality and intensity, pH of the nutrient medium, and agitation and aeration of the culture batch (Kanokwaree and Doran, 1997; Murthy et al., 2014).

Dedifferentiated cells, typically constituting plant cell cultures are usually prone to high variability in product biosynthesis, formation of large cell aggregates, and they are sensitive to shear stress — with the latter two features being particularly important obstacles in industrial-scale cultivation (Yun et al., 2012). Recent research indicates e.g., that undifferentiated cambial meristematic cells can be successfully used for the initiation of plant cell cultures which lack these detriments, are morphologically and physiologically stable, and exhibit high growth rates (Jang et al., 2012; Lee et al., 2010). Cambial meristematic cells are considered to represent a key platform technology for the large-scale production of plant natural products (Roberts and Kolewe, 2010; Yun et al., 2012).

Elicitation is a further strategy to improve the accumulation of secondary metabolites in plant cell and organ cultures. The major function of plant secondary metabolites is the protection from pathogens and insects, or from other biotic or abiotic stresses (Ramakrishna and Ravishankar, 2011; Zhao et al., 2005). Such stress molecules and conditions, which activate plant defense mechanisms, including the synthesis of secondary metabolites, have been termed “elicitors” (Dörnenburg and Knorr, 1995). Biotic elicitors include substances such as pectin, chitosan, methyl jasmonate, or yeast extract, while salicylic acid, heavy metals, or electromagnetic treatment are typical abiotic elicitors (Shilpa et al., 2010). Consequently, elicitation has been successfully utilized for the enhancement of secondary product formation in cell and organ cultures of many plants (Namdeo, 2007; Sharma et al., 2011; Shilpa et al., 2010). The progressive understanding of signal transduction pathways in the elicitor-induced production of secondary metabolites will be important for the further optimization of plant tissue culture-based production systems (Zhao et al., 2005).

Metabolic engineering holds a high potential for the enhancement of biomolecule formation in plant cell cultures. A range of techniques are nowadays available for the identification of rate-limiting gene products within biosynthetic pathways, and the subsequent transient or stable transformation of plant cells with the aim of up- or down-regulating relevant gene products (Wilson and Roberts, 2012). Herein, major progress has been achieved in the manipulation of the biosynthesis of various terpenoids, flavonoids, and alkaloids in a range of plants (Glenn et al., 2013; Mora-Pale et al., 2013).

In general, the productivity of a plant *in vitro* culture is related to the degree of differentiation, and this is one of the reasons why in many cases only low yields of desired secondary metabolites can be found in cell cultures (Kolewe et al., 2008; Sauerwein et al., 1992). In recent years hairy root cultures have received increasing interest. The hairy root disease is the result of the infection of wounded plants by the soil bacterium, *Agrobacterium rhizogenes*. The molecular mechanisms underlying this disease, which can cause significant losses in agriculture, have been largely elucidated and reveal a form of natural genetic engineering (Georgiev et al., 2007; Ono and Tian, 2011). Specifically, the so-called T-DNA containing *rol* genes and harbored by the bacteria's root inducing (Ri) plasmid is transferred to the plant cell and integrated into the nuclear genome. As a result, prolific growth of neoplastic roots occurs, and these hairy root systems can be maintained in *in vitro* culture (Ron et al., 2014). Hairy root cultures have been established from many plant species and have been shown to be a valuable biological system with a wide range of applications such as studies in root physiology, phytoremediation, elucidation of biosynthetic pathways, molecular breeding, and production of secondary metabolites (Ono and Tian, 2011). Hairy roots exhibit high growth rates without the need of phytohormones. Unlike cell cultures they are genetically and biochemically stable, and compared to the respective mother plant they often show the same or even greater biosynthetic capabilities for secondary metabolite production (Georgiev et al., 2012; Guillon et al., 2006; Mora-Pale et al., 2014). As with cell cultures, secondary metabolite production can often be enhanced through optimized growth conditions or by using elicitors, and through metabolic engineering by transferring heterologous genes with engineered *A. rhizogenes* strains (Georgiev et al., 2012). Recent research on the production through hairy roots of lignans (Wawrosch et al., 2014), steroids (Doma et al., 2012), anthraquinones (Huang et al., 2014), or alkaloids (Pandey et al., 2014) clearly indicates that optimized hairy root culture systems can be used for the sustainable biotechnological production of biologically active compounds. Regarding the commercial application, the Swiss company ROOTec Bioactives AG (http://www.rootec.com) has developed an optimized bioreactor system for large-scale cultivation of hairy roots with potential to produce a number of pharmacologically active compounds (Table 6).

### 4.2. Heterologous production

The heterologous biosynthesis of plant-derived natural compounds, i.e. the reconstitution of the target compound's biosynthetic pathway in a foreign host in order to increase product yield, is another alternative to the traditional production routes. This approach has originally been developed in order to transfer the biosynthesis of valuable microbial secondary metabolites from their original producers that are often poorly characterized and difficult to cultivate into microbial hosts that are more amenable to fermentation processes (Ongley et al., 2013; Wenzel and Muller, 2005); however, recently this strategy has also been increasingly applied to plant-derived natural products (Marienhagen and Bott, 2013; Song et al., 2014; Wilson and Roberts, 2014). Predominantly, microbes have served as host organisms, however, heterologous biosynthesis of plant-derived secondary metabolites also has been implemented in plant species genetically more amenable than the native sources, such as tobacco or *Arabidopsis* ssp. (Howat et al., 2014; Miralpeix et al., 2013).

Heterologous biosynthesis has the advantage of being more environmentally friendly than chemical synthesis, since it avoids the use of organic solvents, heavy metals, and strong acids or bases (Marienhagen and Bott, 2013). Furthermore, especially in case of microbial hosts, processes are comparably cost-efficient, as microbial growth is based on inexpensive renewable feedstocks and the fast doubling rates of microorganisms allow short production times. In contrast to synthetic chemistry-based routes, microbial fermentations are readily scalable from the lab bench to industrial-sized fermenters (Chemler and Koffas, 2008; Marienhagen and Bott, 2013). Furthermore, recombinant microorganisms usually do not possess pathways competing to the heterologously expressed one. Therefore, the desired products are chemically distinct and can be rather easily purified without the need to remove closely related constituents (Chemler and Koffas, 2008; Marienhagen and Bott, 2013).

The successful reconstitution of a biosynthetic pathway in a heterologous host requires in-depth knowledge about the involved enzymes and the genes encoding them, its regulation, and its compartmentalization (Miralpeix et al., 2013; Wang et al., 2011b). Therefore, the main obstacles in the establishment of heterologous biosynthesis routes are that for many medicinally important natural compounds, the plant biosynthetic pathways are not fully elucidated and the required pathway genes are not available (Miralpeix et al., 2013; Wang et al., 2011b).

The required genes need to be isolated from their native sources and mobilized into the heterologous host via an appropriate vector construct. Expression strategies depend on the host organism, but will either involve the introduction of plasmids that remain episomal or of constructs that integrate into the genome and become a new genetic locus (Miralpeix et al., 2013).

The choice of the appropriate host system is crucial for success of heterologous production. Concerning microbial hosts, *Escherichia coli* and *Saccharomyces cerevisiae* have been most widely applied. Both are non-pathogenic microorganisms that have been extensively used for industrial fermentation and have served as model organisms for fundamental molecular biology research, resulting in a high level of knowledge on their physiology and genetic manipulation (Chen et al., 2013; Siddiqui et al., 2012; Zhang et al., 2008). However, *E. coli* is unable to perform post-translational modifications, its intracellular capacity for lipophilic compounds is limited, and due to the narrow range of inherently produced secondary metabolites, *E. coli* provides no endogenous precursors required for the biosynthesis of some compound classes, for example originating from the mevalonate pathway that is needed for isoprenoid biosynthesis (Miralpeix et al., 2013; Zhang et al., 2008). Moreover, as a prokaryote, *E. coli* has no intracellular compartments. This complicates the implementation of certain eukaryotic enzymes such as the cytochrome P450s, that include several key enzymes of flavonoid biosynthesis, and that are usually attached to the eukaryotic cell's endoplasmic reticulum (Miralpeix et al., 2013; Wang et al., 2011b). In this respect, *S. cerevisiae* provides several distinct advantages over *E. coli*: as a eukaryote, yeast has intracellular compartments, allowing posttranslational modification of eukaryotic proteins as well as functional expression of membrane-bound cytochrome P450 enzymes (Siddiqui et al., 2012; Wang et al., 2011b).

Also plant-based expression platforms, including transgenic cell suspension cultures, hairy root cultures, and whole plants, have been used for the heterologous biosynthesis of secondary plant metabolites (Miralpeix et al., 2013; Vasilev et al., 2014). For example, a recent work described the discovery of the last missing steps of the secoiridoid biosynthesis pathway in *C. roseus* (L.) G. Don and the expression of the complete pathway in the heterologous host *Nicotiana benthamiana* Domin., thus paving the way for the sustainable biotechnological production of valuable alkaloids (Miettinen et al., 2014). While the production of recombinant proteins through transgenic plant cell cultures is not the topic of this review, it should be noted that a number of proteins are currently in clinical stages of development or, as is the case for human glucocerebrosidase, already on the market (Obembe et al., 2011; Ratner, 2010; Wilson and Roberts, 2012). Furthermore, many valuable secondary products that failed to be produced in microbes or in plant cell/tissue cultures can be produced in intact heterologous plants (Miralpeix et al., 2013). As an example, it is possible to produce artemisinin in transgenic tobacco as discussed in more detail below (Farhi et al., 2011).

Once a biosynthetic pathway has been successfully reconstituted in a heterologous host, it is still not guaranteed that the desired metabolite is produced at satisfactory amounts. High titre production might be hampered by the limited availability of one or more precursors needed for biosynthesis thus creating bottlenecks in the pathway, by insufficient stability of the heterologously expressed enzymes against proteolytic activity, by pathway imbalance due to redistribution of metabolic fluxes, by consumption of the produced metabolites in an endogenous pathway of the heterologous host, or by negative or toxic effects of the heterologously produced metabolites to the host organism (Lo et al., 2013; Ongley et al., 2013; Wang et al., 2011b). Therefore, in order to successfully design a heterologous production process, a number of challenges have to be addressed. This is mainly done by metabolic engineering, i.e. by the optimization of the host organism's enzyme, transport, and regulatory functions (Chemler and Koffas, 2008; Gosset, 2009; Li and Pfeifer, 2014). In order to allow efficient metabolic engineering, scientists increasingly make use of tools from the emerging field of synthetic biology in order to enable balanced expression of pathway genes, to allow spatio-temporal separation of the molecular pathway components, and to optimize genes by directed evolution approaches (Jensen and Keasling, 2014; Li and Pfeifer, 2014). The rational application of these tools is increasingly supported by *in silico* tools for modeling of genome-scale metabolism, pathway search and enumeration, metabolic flux analysis, and pathway ranking (Fernandez-Castane et al., 2014).

Based on their biogenetic origin, plant secondary metabolites can be roughly grouped into three major classes: phenylpropanoids, alkaloids, and terpenoids (Peters and Crouteau, 2004). Heterologous production of plant secondary metabolites from all three classes was recently reviewed elsewhere (Marienhagen and Bott, 2013; Miralpeix et al., 2013; Zhou et al., 2014). Since the currently most successful heterologous production process has been developed for an isoprenoid, namely for a precursor of the sesquiterpene artemisinin, the class of terpenoids will be discussed in more detail in the following paragraphs.

**Fig. 4:**
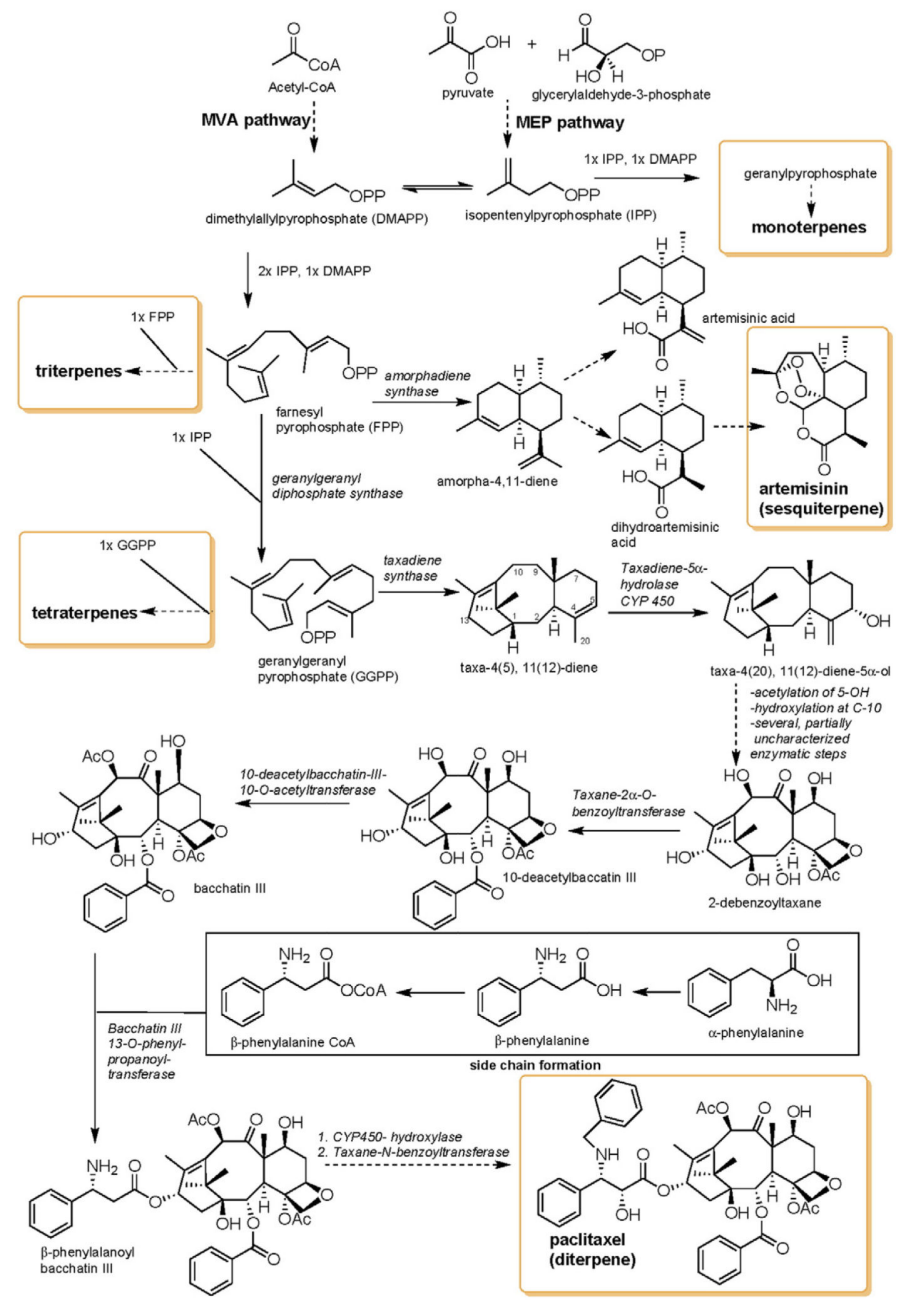
Scheme of the isoprenoid biosynthetic pathway with a focus on the biosynthesis of paclitaxel and artemisinin. Dotted arrows indicate multiple enzymatic steps. The content of the scheme is adapted from: (Lange and Ahkami, 2013; Malik et al., 2011; Paddon and Keasling, 2014).

With at least 40,000 structures known to date, many of them being plant-derived, isoprenoids (also known as terpenoids) represent the largest class of secondary plant metabolites (scheme of the isoprenoid biosynthetic pathway with a focus on the biosynthesis of paclitaxel and artemisinin is presented on Fig. 4). Biosynthesis of plant-derived terpenoids originates from the two isomeric C5-building blocks isopentenyl-pyrophosphate (IPP) and dimethylallyl-pyrophosphate (DMPP). They are formed either in the mevalonate pathway that is compartmentalized in higher plants in the cytosol (early steps), the endoplasmic reticulum (3-hydroxy-3 methylglutaryl-CoA reductase) and peroxisomes (late steps), or from the 2C-methyl-d-erythriol-4-phosphate (MEP) pathway (also known as non-mevalonate pathway) that is present in most prokaryotes, all eukaryotes, and the chloroplasts of higher plants (Kuzuyama and Seto, 2003; Lange and Ahkami, 2013). Condensation of these two C5-pyrophosphates and of larger building blocks derived from them leads to the biosynthesis of monoterpenes (C10, comprising many volatile oil constituents), sesquiterpenes (C15, e.g., artemisinin), diterpenes (C20, e.g., paclitaxel), triterpenes (C30, e.g., the ginsenosides), and tetraterpenes (C40, e.g., the carotenoids) (Bohlmann and Keeling, 2008; Marienhagen and Bott, 2013). Due to their high medicinal relevance and their restricted availability from traditional production routes, very big efforts have been invested in order to facilitate the heterologous production of artemisinin and paclitaxel (Howat et al., 2014; Kong et al., 2013; Lange and Ahkami, 2013; Li and Pfeifer, 2014).

Artemisinin is a sesquiterpene lactone endoperoxide naturally occurring in sweet wormwood (*A. annua* L.). Artemisinin is effective against severe malarial forms and is also currently studied for activity against certain cancer types and viral diseases. However, its availability is limited due to its low abundance in the natural source and due to the low yield of chemical total synthesis, thus resulting in a high price that is unaffordable for people in developing countries where malaria frequently occurs (Kong et al., 2013). Therefore, there has been an intensive exploration of alternative strategies for artemisinin production in order to enhance supply and to reduce costs, among them the development of a heterologous production process. As the artemisinin biosynthetic pathway downstream of artemisinic acid or dihydroartemisinic acid is not yet fully understood, the goal of the hitherto strategies was the heterologous production of artemisinin precursors such as amorpha-4,11-diene, artemisinic acid or dihydroartemisinic acid, followed by their semi-synthetic derivatization to artemisinin (Kong et al., 2013). These efforts have led to the development of an economically feasible production process consisting of heterologous production of artemisinin from inexpensive carbon sources (glucose, ethanol) in a *S. cerevisiae* strain engineered for high yield artemisinic acid production (production titres: up to 25 g/l!), and subsequent semi-synthetic conversion of artemisinic acid to artemisinin by a simple and inexpensive chemical conversion procedure (Paddon et al., 2013). Process development from laboratory research to industrial scale has been funded by a five year grant from the Bill & Melinda Gates Foundation, and involved the University of California, Berkeley, USA, the synthetic biology inventor Amyris Inc., and the non-profit pharmaceutical company One World Health (now PATH's Drug Development Program) (Paddon and Keasling, 2014). Sanofi is now using this technology for large-scale production of artemisinin. The company is committed to apply a no profit, no loss production model in order to maintain a low price for developing countries (http://en.sanofi.com/Images/32474_20130411_ARTEMISININE_en.pdf). Alternatively to this very successful development, efforts have been made to directly produce artemisinin in heterologous plant hosts, since microbial hosts only allowed the heterologous production of artemisinin precursors, while the heterologous production of artemisinin itself could only be realized in plants so far (Farhi et al., 2013). Indeed, Farhi et al. were able to transfer the artemisinin biosynthetic pathway into tobacco plants, using a mega vector construct that consisted of five plant- and yeast-derived genes involved in the mevalonate and artemisinin biosynthetic pathway, all of them regulated by distinct constitutive promoters (Farhi et al., 2011). The gene products were targeted to diverse cellular compartments to increase precursor availability. However, with this approach, the accumulation levels that ranged around 7 μg/g biomass dry weight are currently too low to compete with other production alternatives.

Paclitaxel, a complex natural product with a diterpenoid core, was isolated and structurally elucidated for the first time in the late 1960s from the stem bark of western yew (*T. brevifolia* Nutt.) (Wani et al., 1971). In the late 1970, it was discovered as the first antimitotic agent that acted by promoting the irreversible assembly of tubulin into microtubules (Schiff et al., 1979; Schiff and Horwitz, 1980). Currently, paclitaxel is approved for the treatment of different kinds of cancers, and its fields of application are expected to expand as the compound is currently also studied for the treatment of other, non-cancer related, diseases. Its market price is very high at $ 600,000 per kg (Howat et al., 2014). As extraction from natural sources and total synthesis have proven infeasible to meet the market demands, the compound is currently produced by two different approaches: first, paclitaxel and analogs thereof are produced by semi-synthesis using taxanes that occur more abundantly than paclitaxel in various yew species such as bacchatin III or 10-deacetylbacchatin III as starting materials; second, *Taxus* plant cell cultures are used for paclitaxel production (Howat et al., 2014; Malik et al., 2011). Also heterologous paclitaxel production has been intensively investigated. Indeed, the early-stage paclitaxel intermediate taxadiene could be produced in several microbial and plant heterologous hosts: while production in *Arabidopsis thaliana* (L.) Heynh. only reached levels of 600 ng per gram dry weight (Besumbes et al., 2004), transgenic tomato plants overexpressing taxadiene synthetase and lacking the ability to utilize geranylgeranyl diphosphate (GGPP) for carotenoid biosynthesis accumulated up to 160 μg paclitaxel/g freeze dried tomatoes, which is close to the quantities reported in western yew bark (Kovacs et al., 2007). As both transfected *Arabidopsis* and tomato plants were found to grow more slowly than the wild type plants, Anterola et al. (2009) used the moss *Physcomitrella patens* (Hedw.) Bruch & Schimp. as heterologous taxadiene production platform, and achieved production levels of 0.05% of fresh weight without growth retardation of the host (Anterola et al., 2009). Concerning microbial heterologous hosts, taxadiene levels of 8.7 mg/l could be reached in *S. cerevisiae* (Engels et al., 2008). High-level production of up to 1 g taxadiene/l could be achieved in engineered *E. coli* strains (Ajikumar et al., 2010). However, one has to keep in mind that taxadiene is only a very early-stage intermediate of the paclitaxel biosynthetic pathway (Fig. 4). As an example, it would be necessary to engineer six more hydroxylation reactions and other steps (including several yet unidentified ones) in order to achieve baccatin III, a feasible precursor for paclitaxel semi-synthesis. However, already the introduction of the genes encoding the enzymes to produce taxa-4 (20),11(12)-diene-5α-ol, the intermediate following taxadiene in the paclitaxel pathway, led to a significant reduction of productivity (below 60 mg/l) (Ajikumar et al., 2010). In addition, several steps in the overall pathway have not been conclusively identified or confirmed. Therefore, the total heterologous production of paclitaxel is not within reach to date.

As can be seen from the examples discussed above, heterologous production has the potential to serve as an alternative production route, especially for plant-derived compounds that are difficult to access due to restricted abundance in their natural sources or due to structural constraints that make a synthetic approach unfeasible. However, it is evident that the technique is currently far from being a commonly applicable alternative to traditional production routes of natural compounds.

The major obstacle in the development of heterologous production processes is the current lack of in depth-knowledge concerning the biosynthetic pathways of many medicinally important plant constituents. Therefore, significant efforts will be necessary to achieve complete elucidation of key target pathways in medicinal plants, e.g., the monoterpene indole alkaloid pathway to vinblastine and vincristine in *C. roseus* (L.) G. Don (Miralpeix et al., 2013). As from the 25 plants with completely sequenced genomes published to date, only a few are medicinal plants, more or less all metabolic engineering approaches involving genes from medicinal plants have started with an incomplete datasets. The complete sequencing of representative medicinal plant species would therefore allow more rationally designed metabolic engineering strategies (Miralpeix et al., 2013). This task might be within reach, considering the revolutionary progresses sequencing techniques are currently facing (Shendure and Ji, 2008). In cases where the total heterologous production of a complex natural compound is not (yet) possible, the production of decorated skeletons that are easily accessible to subsequent semi-synthetic derivatization could also be an option, as has been shown in the case of artemisinin.

A further problem is the low yield of many heterologous production processes, that makes them economically unfeasible or not competitive to other, maybe less environmentally-friendly or sustainable approaches. There are many issues to be considered in order to finally allow the heterologous production of an increasing number of plant-derived secondary metabolites in economically feasible processes, including the modification of pathway enzymes to improve their compatibility to the host organisms; the design of more stable expression systems that allow tens of heterologous enzymes to be expressed simultaneously; enhancement of the tolerance of host microorganisms to the heterologously produced metabolites, e.g. by adaptive evolution strategies; and the development of better algorithms for *in silico* model-based pathway design programs in order to allow a rational application of systems biology tools to the complex biosynthetic pathways of secondary plant metabolites (Miralpeix et al., 2013; Zhou et al., 2014).

### 4.3. Organic synthesis

Over nearly 200 years, the synthetic chemistry community has achieved a vast and diverse arsenal of transformations for bond connection and functionalization of organic molecules at an ever increasing pace. Total synthesis of a plethora of intricate naturally occurring compounds, many originating from plants, could be achieved (Nicolaou, 2014), very often as a result of arduous work and sometimes in a race between academic research groups. Yet, these synthetic endeavors are not reflected in the broad industrial manufacture of natural products. There is at least one total synthesis for each of the plant-derived natural products approved for therapeutic use in the last thirty years, presented in Table 1, with exemplary syntheses of (+)-artemisinin (Zhu and Cook, 2012), (+)-arglabin (Kalidindi et al., 2007), (−)-cannabidiol (Petrzilka et al., 1969), capsaicin (Kaga et al., 1989), (−)-colchicine (Lee et al., 1998), dronabinol ((−)-Δ^9^-*trans*-tetrahydrocannabinol (THC); (Trost and Dogra, 2007)), (+)-ingenol (as described in greater detail in the next paragraphs, ingenol is chemically converted to ingenol mebutate for therapeutic supply; (Liang et al., 2012)), masoprocol (*meso*-nordihydroguaiaretic acid; (Gezginci and Timmermann, 2001)), omacetaxine mepesuccinate ((−)-homoharringtonine; (Eckelbarger et al., 2008)), (−)-paclitaxel (Nicolaou et al., 1994), and (−)-solamargine (Wei et al., 2011a). Also found in this list is (−)-galanthamine, which is particularly notable because it is a rather complex plant-derived compound for which an entirely chemical industrial-scale production process exists (as described in detail in the next paragraphs).

Costs and feasibility of reagents and catalysts, exothermic and cryogenic reaction characteristics, chromatographic purification, solvents, spent reagents and byproducts handling are among the aspects that chemical engineering is concerned with during process development of therapeutic compounds (Ager, 2011). Specifically, the safety, health and environment (SHE) considerations led to the formulation of a framework now known as Green Chemistry. This concept was introduced in the early 1990s by Anastas of the US Environmental Protection Agency (EPA, 2002). The key aspect therein is about how to “design (…) chemical products and processes to reduce or eliminate the use and generation of hazardous substances” (Anastas and Warner, 1998; Horvath and Anastas, 2007). It attempts to achieve sustainability at the molecular level and applies to all stages of the chemical life cycle (Anastas and Eghbali, 2010). Key to this are the Twelve Principles of Green Chemistry, meant to be a cohesive set of guidelines for the design of products and processes. Outlined very briefly, they are: Prevention (of waste, rather than treating it), Atom Economy (vide infra), Less Hazardous Chemical Synthesis (applies to methodology), Designing Safer Chemicals (while preserving their intended function), Safer Solvents and Auxiliaries (if they cannot be avoided altogether), Design for Energy Efficiency (methodologies should allow ambient temperature and pressure), Use of Renewable Feedstock (rather than depleting limited raw materials), Reduce Derivatives (in concession steps such as non-strategic redox reactions and functional group interconversions, protective group manipulations), Catalysis (as selective as possible), Design for Degradation (applies to products which should break down to nonhazardous materials at the end of their function), Real-Time Analysis for Pollution Prevention (before hazardous substances are formed), and Inherently Safer Chemistry for Accident Prevention (minimizing the hazard factor in the risk function). These principles are discussed in more detail with examples elsewhere (Anastas and Eghbali, 2010).

Meaningful statements about the impact on health and environment also require a quantitative assessment to describe the various shades of green of a product or process. For this reason, different metrics have been proposed, gradually marking a paradigm shift from the overall yield as the most important criterion toward a more integrated view (Dicks and Hent, 2015). With regard to a synthetic plan (chemical reactions on paper), the concept of atom economy *AE* was proposed early on (Trost, 1991) as a measure of how much of the reactants, in terms of their molecular weight *M*, remains in the desired final product (Eq. (1)).

(1) $$\mathrm{AE}=\frac{M_{\text{product}}}{\sum M_{\text{reactants}}}\times 100\%$$

When molecules are transformed, waste material is produced in most cases, depending on the type of reaction mechanism. For example, addition and rearrangement reactions tend to be more atom economic while substitution and elimination reactions do not. When extending the concept to a reaction sequence composed of a number of individual steps, the intermediate compounds do not appear in Eq. (1) because they cancel from the overall net reaction equation. A reactant is defined as any component which contributes atoms to intermediate or final products, even if none of the reactant eventually remains in the final product as it is the case in protection–reaction–deprotection sequences. Catalytically active species, solvents and other auxiliary components are neglected unless they are (partially) incorporated into intermediate or final products. The equivalents of reaction components used to calculate *AE* are taken as demanded by the stoichiometry of the transformation, i.e. disregarding the excess of some components one may choose to use in a real chemical reaction. The advantage of this approach is that chemists can evaluate different synthetic strategies before real experiments are attempted (Dicks and Hent, 2015), where subsequent reaction optimization then very likely calls for reactant equivalents, catalysts, solvents, auxiliaries, and other conditions to be varied considerably.

For the same reasons, however, atom economy is also quite remote from real-world chemistry. A metric which actually accounts for experimental details, called the reaction mass efficiency (Curzons et al., 2001), also known as Curzons *RME* (Eq. (2)), was defined as the quotient of real product mass by the sum of reactant masses.

(2) $$\text{Curzons}\;\mathrm{RME}=\frac{m_{\text{product}}}{\sum m_{\text{reactants}}}=\mathrm{AE}\times \varepsilon \times \frac{1}{\mathrm{SF}}$$

It is equal to the product of *AE*, experimental yield *ε*, and the inverse of the stoichiometric factor *SF* (as defined in Eq. (3)), the latter being created to account for reactant excess (Andraos, 2005).

(3) $$\mathrm{SF}=1+\frac{\sum m_{\text{excess},\text{reactants}}}{\sum m_{\text{stoichiometric},\text{reactants}}}$$

It was shown that Curzons *RME* could be used to compare costs for drug manufacture in general (Constable et al., 2002). A related quantity is the maximum or kernel *RME* (Andraos, 2005), which applies to a hypothetical scenario where all excess reagent is recovered (i.e. where *SF* equals unity), thus being the best value possible for Curzons *RME* under given yield *ε* (Eq. (4)).

(4) kernelRME=AE×ε

The most critical view of a process is the generalized *RME* (Andraos, 2005) because it is calculated as the mass of product divided by the total mass of all input materials (including catalysts, auxiliaries, solvents and work-up and purification materials, Eq. (5)).

(5) $$\text{generalized}\;\mathrm{RME}=\frac{m_{\text{product}}}{\sum m_{\text{input materials}}}=\mathrm{AE}\times \varepsilon \times \frac{1}{\mathrm{SF}}\times \mathrm{MRP}$$

It relates to Curzons *RME* by the material recovery parameter *MRP* which takes into account masses of collected product, catalyst *c*, reaction solvent *s*, and all other relevant materials *ω* (Eq. (6)).

(6) $$\mathrm{MRP}={\left[1+\frac{\mathrm{AE}\times \varepsilon \times \left(c+s+\omega \right)}{m_{\text{product}}\times \mathrm{SF}}\right]}^{-1}$$

In order to organize all this information, a visual aid called the radial pentagon was proposed (Andraos and Sayed, 2007).

Contrarily to the green metrics discussed so far, the most prominent counter-green metric (the ideal value of which is zero) is the environmental impact factor, or *E* factor, as introduced by Sheldon (Sheldon, 1992), which focuses on waste production irrespective of where it originates from (Eq. (7)).

(7) $$E\;\text{factor}=\frac{\sum m_{\text{input materials}}-m_{\text{product}}}{m_{\text{product}}}$$

Notably, *E* factors associated with pharmaceutical production are generally higher by three to four orders of magnitude when compared with the oil refining petrochemical industry (Sheldon, 2007), and non-optimized laboratory preparations of complex (natural) products have *E* factors which are even far higher.

It is important to understand that no single metric will completely reflect all aspects of efficiency and safety. Moreover, calculating various metric parameters for multistep (especially convergent) synthetic plans by hand is rather impractical, since it is easy to lose track and make mistakes. To address this, a spreadsheet based, step-by-step algorithm was created for practical application to any complex synthesis, and the method was applied to exhaustively compare the greenness of 18 synthetic plans (both industrial and academic) of oseltamivir (Andraos, 2009), the initial preparations of which were based on the elaboration of quinic acid (obtained from e.g. the bark of *Cinchona* species) or shikimic acid (from *Illicium* species, such as the Chinese or Japanese star anise). This revealed that the industrial semi-synthetic shikimic acid route (Harrington et al., 2004) and a streamlined modification thereof (Carr et al., 2008) had the highest overall yield *ε* (39.0% and 43.8%) and the highest *kernel RME* (0.115 and 0.113), while they had the lowest value for the *E*-factor (230.9 and 203.5). The industrial route produced the least amount of total waste (94.7 kg for 1 mol, or 230.8 kg for 1 kg of oseltamivir phosphate). It was also noted that academia continues to improve existing synthesis as well.

Generally, semi-synthesis can provide access to large quantities of a desired drug if a suitable precursor is available. As mentioned in Section 4.2, paclitaxel is an example of this sort. While more than 1400 metric tons of *T. brevifolia* Nutt. (Pacific yew) bark had to be collected in 1991 and 1992 (Cragg, 1998) to supply the compound for clinical trials only, thereby killing the slow-growing trees, the tetracyclic diterpene 10-deacetylbaccatin III (representing the structurally most demanding moiety of paclitaxel) is readily obtainable from the needles of *Taxus baccata* L. (European yew) in 1 g/kg (Guenard et al., 1993). This harvesting method leaves the trees intact, and 10-deacetylbaccatin III is converted chemically to paclitaxel in high yield (Denis et al., 1988; Guenard et al., 1993; Holton, 1990). Thereafter, a chemoenzymatic process was developed in which the chiral intermediate necessary to elaborate 10-deacetybaccatin III to paclitaxel was produced via kinetic resolution employing reusable, immobilized lipases, and the process was scaled to up to 150 l, resulting in excellent yield, enantiomeric excess and purity (Patel et al., 1994).

Strategies for natural product synthesis are not readily delineated by type of chemical reaction, although a methodology-oriented central step may sometimes be identified. Bond metathesis affording a macrocyclic structure is a characteristic example (Fürstner, 2011; Nicolaou et al., 1998), in which case the key transformation occurs toward the end of the synthesis. On the other hand, a synthetic strategy may be built upon procuring important intermediates or starting materials. An example for this (from chemoenzymatic methodology) is the enantioselective dihydroxylation of variously substituted benzenes by toluene dioxygenase (Hudlicky and Reed, 2009). This transformation is remarkable since it accomplishes dearomatization of benzene derivatives to give arene dihydrodiols, allowing for synthetic routes, which would otherwise not be feasible. It is also a whole-cell fermentation (in *E. coli*) which appears well-scalable (up to 15 l of broth in a laboratory setting), furnishing the dihydrodiols on 10 to 100 g scale within three days, including the time needed for preculture preparation and inoculation (Endoma et al., 2002). Compounds of this sort, the production of which by chemical means would be a lengthy procedure, are highly useful building blocks because chemical synthesis might be next used to elaborate them further to plant compounds such as pancratistatin and morphine (Hudlicky, 2006; Reed and Hudlicky, 2015), or analogs and derivatives thereof, and also oseltamivir (Werner et al., 2010), in the order of ten steps. This hand-in-hand of biotransformation and synthesis improves the metrics discussed above in two ways: the enzymatic process itself fulfills most of the Twelve Principles of Green Chemistry very well, except for the Renewables principle because the substituted benzene, although readily prepared chemically, may still originate from a hydrocarbon source, and the Safer Chemicals principle because such dihydrodiols unfortunately can violently rearomatize upon exposure to trace acid, while the Degradation principle would not apply to these intermediates. Additionally, a short overall synthesis is possible because the dihydrodiol already contains important strategic bonds of the target molecule.

Identification of such strategic bonds, however, is a general approach in synthetic planning and applies to all types of strategies and methodologies. Therein, the target molecule is conceptually disconnected (retrosynthesis) into simpler fragments (synthons). This process is iterated until the synthons translate into readily available and inexpensive starting materials for the forward synthesis (Corey and Cheng, 1989). A specific instance of such a disconnection approach in terms of natural products is biomimetic synthesis. The idea is to have at least one key step which retrosynthetically emulates the same bond disconnections as the (proposed) biosynthesis, in an effort to render the route to the final product more efficient. The concept may be traced back to the synthesis of tropinone by Robinson (Robinson, 1917), but the field really gained momentum in the early 1970s (Breslow, 1972). Successful laboratory preparations involving intermediate structures that also occur in natural systems (e.g., plants) have also been used to support biosynthetic rationales. Thus, biomimetic synthesis (also called bioinspired synthesis) has given rise to numerous natural products, as reviewed by Bulger et al. (Bulger et al., 2008).

Collective total synthesis represents a more recent concept related to biomimicry and the idea of modularity in synthesis that focuses on generating structurally diverse natural products (Jones et al., 2011b), as demonstrated with different alkaloids belonging to the *Strychnos, Aspidosperma* and *Kopsia* families by pointing out their presumably common biosynthetic precursor preakuammicine (Hudlický and Reed, 2007). This led to the preparation of a variety of such alkaloids via a key intermediate structure, obtained in an organocatalytic and enantioselective cascade in high yields. Notably, this approach also constitutes the most concise synthesis of (−)-strychnine as the subject of many total syntheses (Bonjoch and Sole, 2000; Cannon and Overman, 2012) to date, with an overall yield of 6.4% in 12 steps starting from a commercially available compound. This compares to 2 × 10^−4^% overall and 29 steps in the landmark Woodward synthesis of the compound (Woodward et al., 1954). This collective synthesis approach for plant compounds has subsequently been taken up by others (Ghavimi and Magnus, 2014; Han et al., 2014; Li et al., 2013).

Scalability of chemical reactions is a concern in its own right. The mass and heat transfer rates change dramatically when scaling to a large reactor, affecting reaction rate and selectivity. For example, reactions such as lithiations are typically carried out at −78 °C (the sublimation point of dry ice), but on industrial scale, the increased viscosity and low solubility of such a solution can lead to inhomogeneity and possibly impurity formation. In the downstream process, problems include large-scale liquid–liquid extraction (formation of emulsions) and the need to tightly control the crystallization process (polymorphism and solvate formation) of every batch (Laird, 2010; Stitt and Simmons, 2011). Moreover, the very nature of the step-by-step process requires many manual operations. Most work-up und purification procedures generate waste and consume energy, making alternatives where production processes are telescoped highly desirable. Continuous flow chemistry holds the potential of bypassing scaling problems and improving the environmental footprint. Considerable efforts have been invested into managing multi-step processes, downstream processing and handling low temperatures, gases and slurries. This is combined with increased process safety, real-time reaction monitoring and control, and the potential to reduce solvent amounts and waste production, as well as truly continuous (i.e., 24/7) operation (Pastre et al., 2013), well in agreement with the Twelve Principles. For example, a flow reactor system has been used to react singlet oxygen with the endocyclic double bond of dihydroartemisinic acid, eventually furnishing artemisinin via a hydroperoxy intermediate with the capability of producing the antimalarial drug at a rate of 200 g per day (Levesque and Seeberger, 2012). Although this method has been criticized in favor of a batch process (Paddon et al., 2013), it has been pointed out that accumulation of this hydroperoxy intermediate in batch might be hazardous (Booker-Milburn, 2012), whereas it is continuously converted further in the flow process. Moreover, flow chemistry is not restricted to semi-synthesis. Preparation of the neolignan grossamide represents a pioneering case as a proof of concept (Baxendale et al., 2006b). There are several other instances of plant-derived compounds being synthesized by the use of flow methods, including (*rac*)-oxomaritidine (Baxendale et al., 2006a), (−)-dumetorine [alongside with the synthesis of the structurally similarly (−)-sedamine and (+)-sedridine (Riva et al., 2011)], and (+)-pauciflorol F (Kim et al., 2011). The rapidly emerging area of flow chemistry-mediated preparation of natural products has been recently discussed in substantial detail (Pastre et al., 2013), and its connections to Green Chemistry (choice of solvents and solvent reduction by an order of magnitude, process safety, recovery and reuse of immobilized reagents, more efficient energy transfer and shortened process time) have been reviewed in detail elsewhere (Ley, 2012).

It has been argued that, once a (single-digit) gram-scale synthesis of a complex natural product has been achieved, it is more likely to be amendable to larger scale without complete revision of the synthetic plan (Kuttruff et al., 2014). The preparation of such quantities, including many plant-derived compounds, mostly represents the state of the art within the constraints of an academic setting (Hong, 2014; Kuttruff et al., 2014). On the other hand, chemical synthesis of plant compounds has been regarded very difficult and expensive due to their structural complexity (De Luca et al., 2012), and there is no consensus on whether synthetic chemistry or synthetic biology will be the major supply route of the future for the production of chemical entities in general, especially in light of recent advances in chemical biology (Keasling et al., 2012). In addition, progress in both areas could very well be used synergistically, as was pointed out above for chemoenzymatic production. Chemical synthesis has reached a level of complexity to achieve a large diversity of bond-formation processes, which can certainly be implemented on industrial scale employing favorable economics for the production of compounds that are available from natural sources in minute quantities only. To this end, a brief case study shall be presented which compares the chemical routes of (−)-galanthamine and (+)-ingenol, two plant-derived and therapeutically used compounds.

**Fig. 5:**
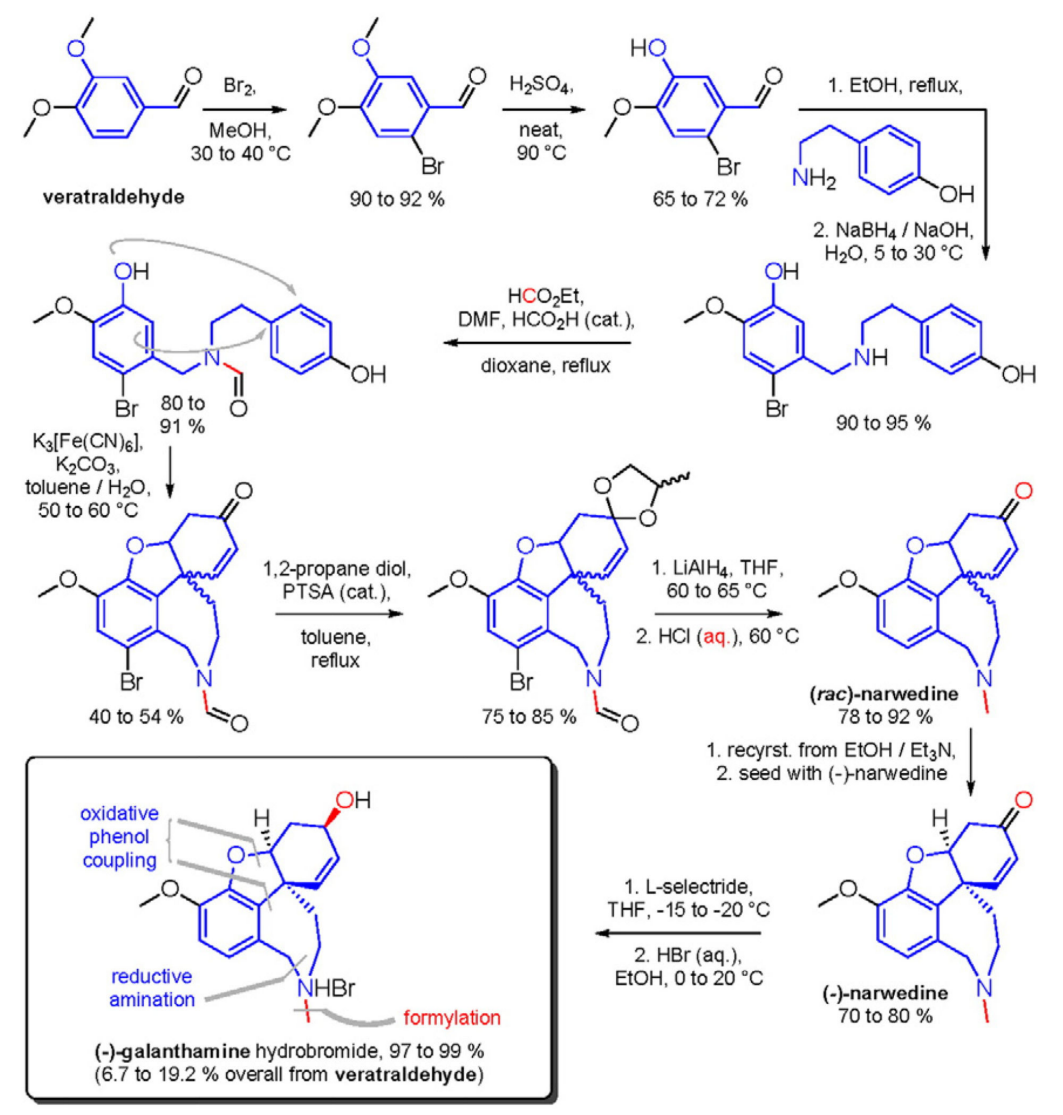
Synthesis of galanthamine. The colors indicate: blue (skeletal atoms/bonds: carbon/oxygen/nitrogen framework), and red (installed non-skeletal atoms/bonds).

Galanthamine (Reminyl^®^ and other trade names, Sanochemia Pharmazeutika) is an Amaryllidaceae alkaloid, and its main use is to alleviate the symptoms of Alzheimer's disease by inhibiting the enzyme acetylcholinesterase to counteract decreased levels of the neurotransmitter in the brain (Jones et al., 2011a). It can be produced by isolation from various *Galanthus* (snowdrop) species, but these sources do not meet the demand, in particular due to highly specific conditions for culturing. Various syntheses had been reported [first by Barton and Kirby (Barton and Kirby, 1962)] before a kilogram scale route was developed to allow the industrial production of galanthamine hydrobromide by chemical means [Fig. 5; (Czollner et al., 2000; Küenburg et al., 1999)].

**Fig. 6:**
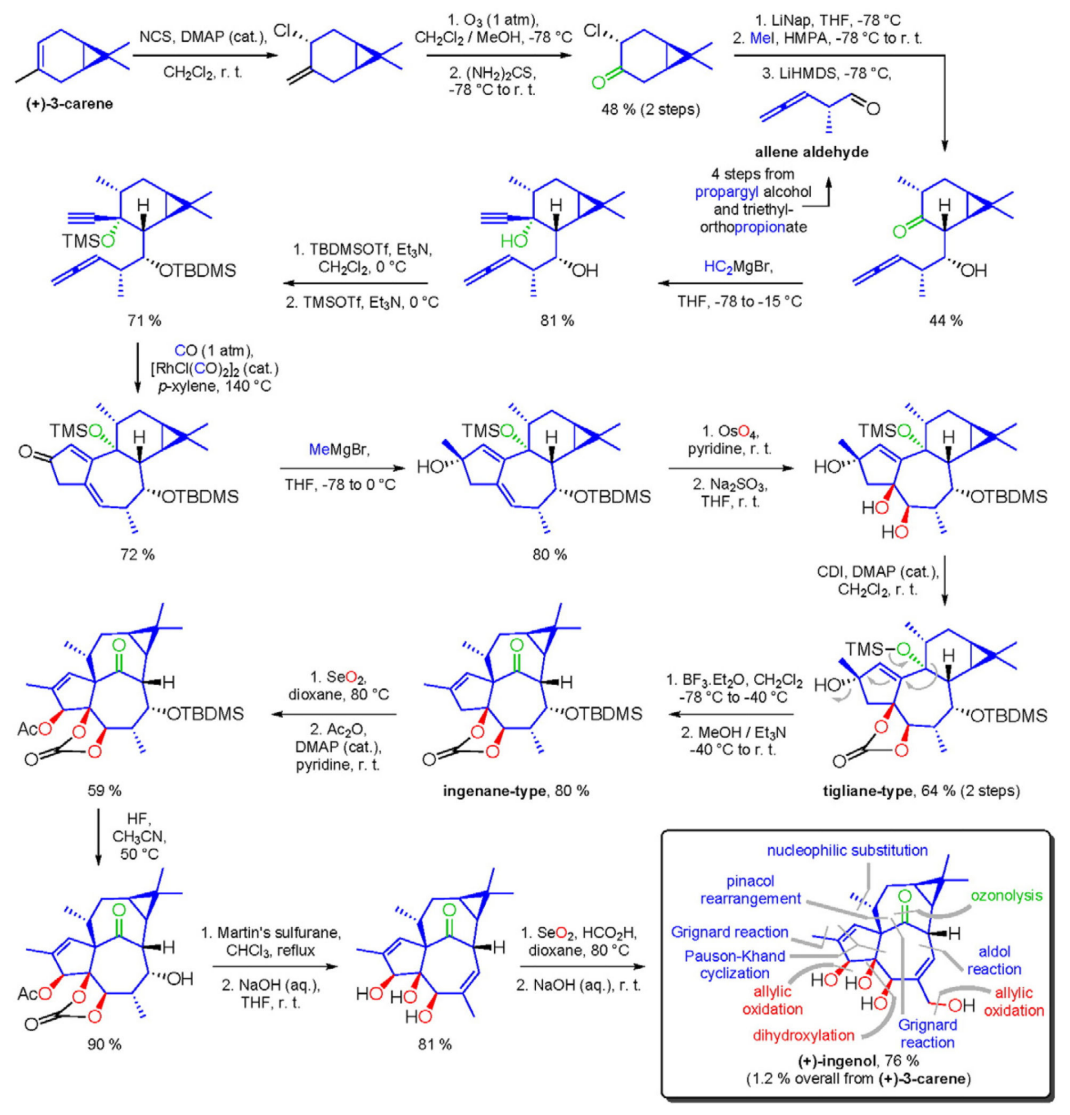
Synthesis of ingenol. The colors indicate: blue (skeletal atoms/bonds: carbon-only framework from cyclase phase), red (installed non-skeletal atoms/bonds: hydroxy functionality set up during oxidase phase), and green (installed non-skeletal atoms/bonds: functionality set up outside oxidase phase).

Ingenol is a diterpenoid found in *Euphorbia* (spurge) species, and most important with regard to its bioactivity is that the naturally occurring derivative ingenol mebutate has recently been approved by FDA and EMA as the first-in-class drug to treat actinic keratosis (Gras, 2013). As ingenol mebutate is isolated from *E. peplus* L. in yields of only 1.1 mg/kg (Hohmann et al., 2000), it is currently prepared semi-synthetically from the more abundant parent ingenol [Picato^®^, LEO Pharma; (Liang et al., 2012)], obtained from dried seeds of *E. lathyris* L. in 275 mg/kg (Appendino et al., 1999). This process is still not optimal, and because knowledge on the biosynthesis is scarce, advances on the part of synthetic chemistry were prompted to render ingenol more readily accessible. Importantly, these efforts were also driven by the possibility to explore and produce analogs of this compound with enhanced pharmacological properties. Three total syntheses had been reported previously [as reviewed by (Kuwajima and Tanino, 2005)], but an approach offering a perspective for large scale synthesis was reported only recently [Fig. 6; (Jørgensen et al., 2013; McKerrall et al., 2014)], having elicited considerable interest (Appendino, 2014).

Among other structural features regarding the two compounds discussed in detail here, ingenol has more stereocenters than galanthamine (8 vs. 3), and more non-aromatic quaternary carbons (3 vs. 1), two of which are contiguous. In the case of ingenol, asymmetry is introduced by using the naturally occurring and readily available (+)-3-carene as a starting material, with the remainder of the synthesis relying on substrate-controlled diastereoselective reactions with non-chiral reagents only. By contrast, access to optically active galanthamine is achieved because the intermediate narwedine can racemize in solution and be selectively induced to precipitate to enantiomerically pure crystals by seeding with (−)-narwedine, enabling an asymmetric route without any chirality-conferring starting materials or reagents at all. As for the overall synthesis, the most apparent differences are the number of steps (14 + 4 vs. 9), and the use of relatively simple and inexpensive reactants and bulk chemicals for galanthamine compared to the ingenol route. The chemistry is more complex in the latter case, in part because ingenol is entirely carbocyclic, requiring more elaborate methods for bond formation, while the galanthamine synthesis relies mostly on the natural polarities of carbon-heteroatom bonds to furnish new ones. While there are 3 skeleton-forming bond connections (of which one is carbon-carbon during the central oxidative phenol coupling) for galanthamine (Fig. 5), there are 7 such connections for ingenol (all of which are carbon–carbon) during what is called the cyclase phase of the synthesis, in addition to a carbon–carbon bond formation to furnish the allene aldehyde (Fig. 6). Moreover, a key vinylogous pinacol rearrangement to the ingenane-type intermediate and the installment of 4 hydroxyl groups during the oxidase phase take place. In connection with this, and more on the technical side, the use of cryogenic and inert reaction conditions, as well as the air-sensitivity of the reagents, are more pronounced in the ingenol synthesis. Stoichiometric use of osmium tetroxide is also prohibitively expensive and, along with selenium dioxide, a very toxic reagent of the synthesis. More protective group manipulation is also involved, although this is still moderate given the complexity of the molecule. Conducted on kilogram-scale, chromatography is completely absent from the galanthamine synthesis, whereas it is the major purification method in the ingenol synthesis, for which the delivery of the final product on milligram-scale was published. However, key ingenol intermediates were reported to have been produced near the kilogram level in current up-scaling efforts (Drahl, 2013). Although the routes for these two compounds use rather different methodologies, they both mimic the biosynthesis that occurs in snowdrops (Eichhorn et al., 1998) and spurges (Kirby et al., 2010), respectively.

In summary, galanthamine is an example of a plant-derived natural compound which has been produced synthetically on industrial scale for over a decade. The recent work on ingenol, the other example discussed here, stands out because it represents one of the most concise synthetic routes for any natural product of such high complexity expected to be produced synthetically in the future.

## 5. Concluding remarks

Medicinal plants have historically been a rich source for successful drugs, and still represent an important pool for the identification of new pharmacological leads today. Renewed scientific interest in plant-derived natural product-based drug discovery is evident from the analysis of PubMed publications trends (Fig. 1). Plants are producing numerous chemically highly diverse secondary metabolites which are optimized for exerting biological functions and are still far from being exhaustively investigated. Resulting from the revived scientific interest in natural product-based drug discovery, new approaches for the identification, characterization, and resupply of natural products are being developed, that may address some of the challenges related with the development of plant-based therapeutics. One major asset of medicinal plant-based drug discovery is the existence of ethnopharmacological information providing hints for compounds therapeutically effective in humans. In order to harvest its full potential, of particular importance is the adoption of a broad interdisciplinary approach involving ethnopharmacological knowledge, botany, phytochemistry, and more relevant pharmacological testing strategies (e.g., early *in vivo* efficacy studies and compound identification strategies including metabolism and synergistic action of the plant constituents). Resupply from the original plant species is very often unfeasible to meet market demands upon commercialization of a natural product, and alternative resupply approaches are being developed that rely on biotechnological production or chemical synthesis. Total chemical synthesis is an effective resupply strategy in case of natural products or natural product derivatives with simple structures such as acetylsalicylic acid and ephedrine. For complex structures with multiple chiral centers, however, total synthesis is, at present, both difficult and economically unfeasible in most cases, requiring significant technological advances to be successfully applied. For the resupply of complex natural products usually harvesting from plant sources and semi-synthesis from naturally occurring precursors still remain the most economically-viable approaches. Although biotechnological production is currently not broadly applied for industry-scale production of plant-derived natural products, it bears potential that can be harvested in the future align with the progress of the knowledge of plant biosynthetic pathways and the development of more efficient genetic engineering strategies and tools.

While natural product-based drug discovery and development represents a complex endeavor demanding a highly integrated interdisciplinary approach, the presented recent scientific developments, technologic advances, and research trends clearly indicate that natural products will be among the most important sources of new drugs also in the future.

## Figures and Tables

**Table 1:** Plant-derived natural products approved for therapeutic use in the last thirty years (1984–2014)^a^.

Generic name and chemical structure	Plant species (literature reference)	Trade name (year of introduction)	Indication (mechanism of action)
Artemisinin 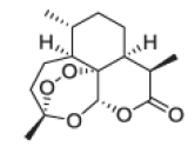	*Artemisia annua* L. (Klayman et al., 1984)	Artemisin (1987)	Malaria treatment (radical formation)
Arglabin 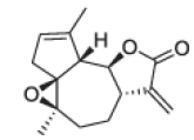	*Artemisia glabella* Kar. et Kir. replaced by *Artemisia obtusiloba* var. *glabra* Ledeb. (Adekenov et al., 1982)	Arglabin (1999)	Cancer chemotherapy (farnesyl transferase inhibition)
Capsaicin 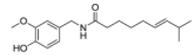	*Capsicum annum* L., or *C. minimum* Mill. (Toh et al., 1955)	Qutenza (2010)	Postherpetic neuralgia (TRPV1 activator)
Colchicine 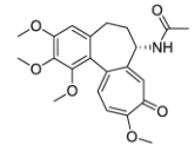	*Colchicum* spp. (Leete and Nemeth, 1960)	Colcrys (2009)	Gout (tubulin binding)
Dronabinol / Cannabidol Dronabinol 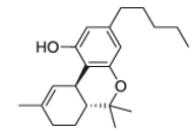	*Cannabis sativa* L. (Vree et al., 1972)	Sativex^b^ (2005)	Chronic neuropathic pain (CB1 and CB2 receptor activation)
Cannabidol 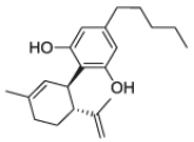			
Galanthamine 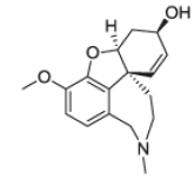	*Galanthus caucasicus* (Baker) Grossh. (Tsakadze et al., 1969)	Razadyne (2001)	Dementia associated with Alzheimer's disease (ligand of human nicotinic acetylcholine receptors (nAChRs))
Ingenol mebutate 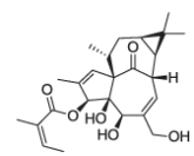	*Euphorbia peplus* L. (Hohmann et al., 2000)	Picato (2012)	Actinic keratosis (inducer of cell death)
Masoprocol 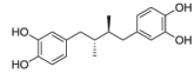	*Larrea tridentata* (Sessé & Moc. ex DC.) Coville (Luo et al., 1998)	Actinex (1992)	Cancer chemotherapy (lipoxygenase inhibitor)
Omacetaxine mepesuccinate (Homoharringtonine) 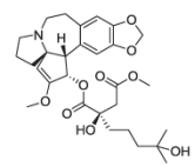	*Cephalotaxus harringtonia* (Knight ex Forbes) K. Koch (Powell et al., 1974)	Synribo (2012)	Oncology (protein translation inhibitor)
Paclitaxel 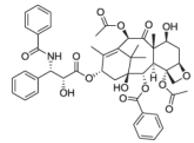	*Taxus brevifolia* Nutt. (Wani et al., 1971)	Taxol (1993), Abraxane^c^ (2005), Nanoxel^c^ (2007)	Cancer chemotherapy (mitotic inhibitor)
Solamargine 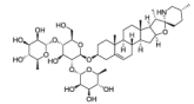	*Solanum* spp. (Hsu and Tien, 1974; Liljegren, 1971)	Curaderm^d^ (1989)	Cancer chemotherapy (apoptosis triggering)

Notes:

a Resources: (Butler, 2005, 2008; Butler et al., 2014; Fürst and Zündorf, 2014; Newman and Cragg, 2012) and www.drugs.com.

b Mixture of the two compounds.

c Paclitaxel nanoparticles.

d Containing not just solamargine but also other solasodine glycosides.

**Table 2:** Plant-derived natural products in clinical trials^a^.

Generic name and chemical structure	Plant species (literature reference)	Number of recruiting clinical trials^b^: indications (*potential mechanism of* *action*)
β-Lapachone 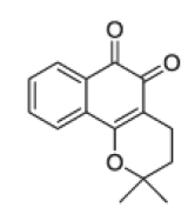	*Haplophragma adenophyllum* (Wall. ex G. Don) Dop (Joshi et al., 1979)	1 trial: Solid tumors (*E2F1 pathway activator*)
Curcumin 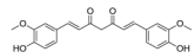	*Curcuma longa* L. (*Turmeric*) (Janaki and Bose, 1967)	26 trials: Cognitive impairment, different types of cancer, familial adenomatous polyposis, schizophrenia, cognition, psychosis, prostate cancer, radiation therapy, acute kidney injury, abdominal aortic aneurysm, inflammation, vascular aging, bipolar disorder, irritable bowel syndrome, neuropathic pain, depression, somatic neuropathy, autonomic dysfunction, Alzheimer's disease, plaque psoriasis, fibromyalgia, cardiovascular disease (*NF-κB inhibition*)
Epigallocatechin-3-*O*-gallate 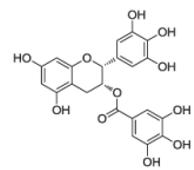	*Camellia sinensis* (L.) Kuntze (*Green tea*) (Kada et al., 1985)	14 trials: Epstein-Barr virus reactivation in remission patients with nasopharyngeal carcinoma, multiple system atrophy, Alzheimer's disease, cardiac amyloid light-chain amyloidosis, Duchenne muscular dystrophy, cystic fibrosis, diabetic nephropathy, hypertension, fragile X syndrome, different types of cancer, obesity, influenza infection (*cell growth arrest and apoptosis induction*)
Genistein 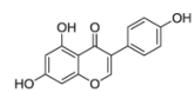	*Genista tinctoria* L. (Perkin and Newbury, 1899)	5 trials: Colon cancer, rectal cancer, colorectal cancer, Alzheimer's disease, non-small cell lung cancer, adenocarcinoma, osteopenia, osteoporosis (*protein-tyrosine kinase inhibitor, antioxidant*)
Gossypol 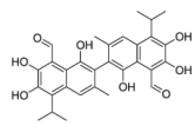	*Gossypium hirsutum* L. (Heinstein and El-Shagi, 1981)	2 trials: B-cell chronic lymphocytic leukemia, refractory chronic lymphocytic leukemia, stage III chronic lymphocytic leukemia, stage IV chronic lymphocytic leukemia, non-small cell lung cancer (*Bcl-2 inhibitor*)
Picropodophyllotoxin 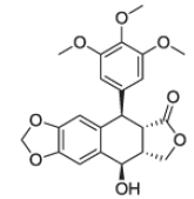	*Podophyllum hexandrum* Royle, replaced by *Sinopodophyllum hexandrum* (Royle) T.S. Ying (Jackson and Dewick, 1984)	1 trial: Glioblastoma, gliosarcoma, anaplastic astrocytoma, anaplastic oligodendroglioma, anaplastic oligoastrocytoma, anaplastic ependymoma (*tubulin binding/IGF-1R Inhibitor*)
Quercetin 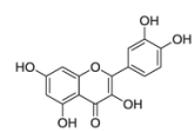	*Allium cepa* L. (Bilyk et al., 1984)	9 trials: Chronic obstructive pulmonary disease, Fanconi anemia, different types of prostate cancer, diabetes mellitus, obesity, diastolic heart failure, hypertensive heart disease, heart failure with preserved ejection fraction, hypertension, oxidative stress, Alzheimer's disease, pancreatic ductal adenocarcinoma, plaque psoriasis (*NF-κB inhibition*)
Resveratrol 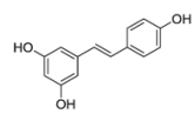	*Vitis vinifera* L. (Langcake and Pryce, 1976)	22 trials: Pre-diabetes, vascular system injuries, lipid metabolism disorders (including non-alcoholic fatty liver disease), endothelial dysfunction, gestational diabetes, cardiovascular disease, type 2 diabetes mellitus, inflammation, insulin resistance, disorders of bone density and structure, metabolic syndrome, coronary artery disease, obesity, memory impairment, mild cognitive impairment, diastolic heart failure, hypertensive heart disease, heart failure with preserved ejection fraction, hypertension, oxidative stress, polycystic ovary syndrome, Alzheimer's disease (*NF-κB inhibition*)

Notes:

a Resources: (Butler, 2005, 2008; Butler et al., 2014; Fürst and Zündorf, 2014; Newman and Cragg, 2012), www.clinicaltrials.gov, and www.drugs.com.

b Determined from www.clinicaltrials.gov on 22nd of October, 2014, including trials in which the respective natural product is applied alone or as a mixture with other constituents, excluding studies with unknown status.

**Table 3:** Approaches to select plant material for natural product drug discovery.

Approach	Characteristics	Examples
Random approach	Random selection of extracts from different plant species, enriched fractions, or isolated natural products, mainly on the basis of their availability.	Gyllenhaal et al. (2012), Khafagi and Dewedar (2000), Nielsen et al. (2012), Oliveira et al. (2011), Shaneyfelt et al. (2006), Spjut (1985), Yuan et al. (1991)
Ethnopharmacological approach	Selection of the test samples based on traditional medicinal applications of the plant species.	Atanasov et al. (2013a), Ekuadzi et al. (2014), Fakhrudin et al. (2014), Noreen et al. (1998), Siriwatanametanon and Heinrich (2011)
Chemosystematic approach	Selection of the test samples based on chemotaxonomy and phylogeny taking into account that plant species from some genera or families are known to produce compounds or compound classes associated with a certain bioactivity or therapeutic potential.	Alali et al. (2005), Alali et al. (2008), Cook et al. (2014), Gunawardana et al. (1992), Harinantenaina et al. (2008), Prasain et al. (2001), Rønsted et al. (2008)
Ecological approach	Selection of test samples based on the interactions between organisms and their environment, considering that plant secondary metabolites possess ecological functions from which a potential therapeutic use for humans can be derived.	Brantner et al. (2003), Coley et al. (2003), Egan and van der Kooy (2012), Krief et al. (2006), Mans et al. (2000), Nacoulma et al. (2013), Obbo et al. (2013), Thoppil et al. (2013)
Computational approach	Selection of test samples relying on *in silico* bioactivity predictions for constituents of certain plant species.	Atanasov et al. (2013b), Fakhrudin et al. (2010), Grienke et al. (2014), Grienke et al. (2011), Guasch et al. (2012), Pfisterer et al. (2011), Sathishkumar et al. (2013), Waltenberger et al. (2011), Zhao and Brinton (2005)

**Table 4:** Basic types of bioassays for testing of phytochemicals and their comparative advantages and disadvantages.

Type of bioassay	Advantages	Disadvantages
*In vitro* assays with purified proteins	High throughput; no cell culture or animal facilities necessary.	Prone to irrelevant hits (compounds with low bioavailability unable to reach the respective target in intact cells or *in vivo*).
*In vitro* cell-based target-oriented assays	Medium- to high-throughput; demonstrate efficacy of the hits at the cellular level; the affected molecular target is known, saving further work for mechanism of action studies.	Require access to cell culture facility; do not assure efficacy *in vivo* (e.g., identified hits may not reach their site of *in vivo* action, for example as a result of rapid catabolism in the liver).
*In vitro* phenotypic cell-based assays	Medium- to high-throughput; demonstrate efficacy of the hits at the cellular level; useful for addressing the underlying mechanism of action, whereby such investigations might lead to the discovery of new molecular targets or pathways affecting the respective phenotype.	Require access to cell culture facility; great effort might be needed to identify the affected molecular target(s) underlying the changed phenotype; do not assure efficacy *in vivo* (e.g., identified hits may not reach their site of *in vivo* action, for example as a result of rapid catabolism in the liver).
*In situ / ex vivo* assays with isolated tissues or organs	High pathophysiological relevance; some of the applications allow reduction of the number of used animals and offer higher throughput in comparison to rodent models.	Lower throughput in comparison to cell-based assays; ethical concerns related to the use of animals; short *ex vivo* half-life of the isolated tissues and organs.
*In vivo* rodent models	High pathophysiological relevance demonstrating activity of hits on the level of a whole organism; reasonably high homology in genomes and similarity in physiology to humans; possibility to generate transgenic models.	Low throughput; ethical considerations; need access to an animal facility; require higher amount of the tested substances; possibility of existence of species-related differences (the observed effects might not be relevant for humans); require a great amount of follow-up work to identify the affected molecular targets.
*In vivo* models in zebrafish and *C. elegans*	Medium- to high-throughput due to the possibility to implement automation; pathophysiological relevance due to pharmacological testing in a whole organism; possibility to generate transgenic models; lower price compared to rodent models; requires lower amount of the tested substances in comparison to rodent models.	Increased possibility of species-related differences (the observed effects might not be relevant for humans); ethical considerations; require a great amount of follow-up work to identify the affected molecular target.

**Table 5:** Strategies to identify bioactive compounds from plant extracts.

Strategy	Characteristics	Examples
Bioactivity-guided fractionation	Consecutive fractionation cycles coupled with bioactivity testing in order to gradually enrich the active compounds and finally isolate the pure active principles.	Atanasov et al. (2013a), Fakhrudin et al. (2014), Mabona et al. (2013), Nievergelt et al. (2010), Pathmasiri et al. (2005), Xiao et al. (2007), Zhang et al. (2001)
Metabolic profiling approach	Comprehensive qualitative and quantitative metabolite analysis. Correlation with bioactivity data allows early stage dereplication (identification of already known bioactive constituents). Can also reveal potential synergistic effects.	Gulcemal et al. (2013), Hou et al. (2010), Inui et al. (2012), Keerthi et al. (2014), Mao et al. (2012), Modarai et al. (2010), Sandasi et al. (2012)
Direct phytochemical isolation	Isolation and identification of plant constituents without immediate evaluation of bioactivity. The focus is set on a comprehensive chemical characterization of the plant extract and isolation of novel natural products.	Appendino et al. (2009), Conrad et al. (2004), Deng et al. (2014), Gao et al. (2011), Guo et al. (2013), Ramasamy and Saraswathy (2014), Su et al. (2005), Wiedenfeld et al. (2007)
Synergy-directed fractionation	Similar to the bioactivity-guided fractionation; the generated fractions and compounds, however, are also tested for synergistic interactions. This approach aims to identify synergistically interacting natural products which could be missed with the traditional bioactivity-guided fractionation.	Junio et al. (2011), Ndhlala et al. (2013), Nitteranon et al. (2011), Tafesh et al. (2011), Wang et al. (2014a)
Metabolism-directed approach	An approach directed toward the identification of potential bioactive metabolites, which might not be present in the starting plant material but are formed as a result of metabolic transformation by the body or by intestinal microorganisms.	Actis-Goretta et al. (2012), Akao et al. (2002), Atkinson et al. (2004), Chen et al. (2012), Guerrero et al. (2013), Ling et al. (2012), Wan et al. (2013), Xie et al. (2011)

**Table 6:** Examples of hairy root cultures fromplants producing important pharmacologically active molecules developed from ROOTec bioactives Ltd^a^.

Plant species; examples of bioactive constituents in plants	Appearance of the plant species (left) and the hairy roots (right)
*Atropa belladonna* L. (deadly nightshade); atropine	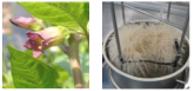
*Nicotiana glauca* Graham (tree tobacco); vitamin D3 derivative, alkaloids	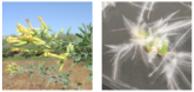
*Nicotiana tabacum* L. (tobacco); anabasine, nicotine, nor-nicotine	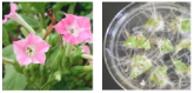
*Ophiorrhiza mungos* L. (mongoose plant); camptothecin	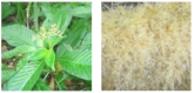

Notes:

a Resource: http://www.rootec.com.

## Abbreviations

4CL: 4-coumaroyl CoA ligase
ADME/T: absorption, distribution, metabolism, excretion (and toxicity)
BIA: benzylisoquinoline alkaloid
C4H: cinnamate 4-hydroxylase
CoA: coenzyme A
CRISPR/Cas9: clustered regulatory interspaced short palindromic repeat/CRISPR associated protein 9
DMPP: dimethylallyl-pyrophosphate
DNP: Dictionary of Natural Products
DNTI: Drugs from Nature Targeting Inflammation
EMA: European Medicines Agency
FDA: US Food and Drug Administration
GGPP: geranylgeranyl diphosphate
GPCR: G-protein coupled receptor
GC: gas chromatography
HPLC: high performance liquid chromatography
HTS: high-throughput screening
iNOS: inducible nitric oxide synthase
IPP: isopentenyl-pyrophosphate
MEP: 2C-methyl-d-erythriol-4-phosphate
MS: mass spectrometry
NCI: (US) National Cancer Institute
NCT: National Clinical Trial
NME: new molecular entity
NMR: nuclear magnetic resonance
NPD: Natural Product Database
OPLS-DA: orthogonal projection to latent structures discriminant analysis
PAL: phenylalanine ammonia lyase
PLS-DA: partial least square regression modeling discriminant analysis
PPAR: peroxisome proliferator-activated receptor
QSAR: quantitative structure activity relationship
TAL: tyrosine ammonia lyase
TALEN: transcription activator-like effector nuclease
TCM: traditional Chinese medicine
THC: tetrahydrocannabinol
SHE: safety, health, and environment
